# The causal association between body composition and 25-hydroxyvitamin D levels: A bidirectional Mendelian randomization analysis

**DOI:** 10.1097/MD.0000000000040618

**Published:** 2024-12-13

**Authors:** Li Jixin, Wenru Wang, Linjie Qiu, Yan Ren, Meijie Li, Wenjie Li, Jin Zhang

**Affiliations:** a Xiyuan Hospital, China Academy of Chinese Medical Sciences, China.

**Keywords:** 25(OH)D, body composition, causality, Mendelian randomization, Obesity, SNP

## Abstract

Observational studies and meta-analyses have indicated a notable correlation between obesity and vitamin D deficiency, yet the causal relationship between the 2 remains contentious. This study employed a bidirectional Mendelian randomization (MR) analysis to explore the interrelation between obesity-associated body metrics: specifically body mass index (BMI), waist-to-hip ratio (WHR), body fat percentage (BFP), whole-body fat percentage (WHF), and 25-hydroxyvitamin D (25(OH)D) levels. Instrumental variables for BMI, WHR, BFP, whole body fat mass (WFM), and 25(OH)D were carefully selected based on predefined thresholds. The association between these metrics and 25(OH)D levels was assessed using the TwoSampleMR package in R 4.2.3. Analysis methods included inverse variance weighted (IVW), MR Egger regression, weighted median, weighted mode, and simple mode. Sensitivity analyses were conducted employing the TwoSampleMR and MR-PRESSO software packages in R 4.2.3 to evaluate heterogeneity and multiplicity of findings. All 4 body components exhibited statistically significant causal associations with decreased 25(OH)D levels: BMI (IVW: odds ratio [OR] = 0.912, 95% confidence interval [CI]: 0.888–0.937, *P* < .001), WHR (IVW: OR = 0.927, 95% CI: 0.882–0.975, *P* = .003), BFP (IVW: OR = 0.883, 95% CI: 0.867–0.899, *P* < .001), and WFM (IVW: OR = 0.850, 95% CI: 0.829–0.872, *P* < .001). However, no statistically significant inverse causative association was observed between these body components and 25(OH)D levels. Sensitivity analyses revealed no substantial heterogeneity or pleiotropy, ensuring robustness of the findings. This study substantiates a significant causal link between 4 obesity-related body components and decreased 25(OH)D levels, excluding reverse causality.

## 1. Introduction

The prevalence of obesity has unequivocally surged globally, evidenced by a doubling of obesity rates in over 73 countries since 1980.^[1]^ Obesity stands as a pivotal risk factor for a spectrum of diseases including type 2 diabetes, nonalcoholic fatty liver disease, myocardial infarction, and hypertension.^[2]^ The World Obesity Federation, in collaboration with the American and Canadian Medical Associations, emphasizes that obesity is not solely a disease but rather a multifaceted contributor to various diseases, presenting an urgent global health burden demanding resolution.^[3]^ Vitamin D, a fat-soluble nutrient pervasive in human tissues and cells, plays a pivotal role in maintaining extracellular calcium and phosphate levels, regulating bone metabolism, and ensuring calcium balance.^[4]^ Its conversion in the liver to 25-hydroxyvitamin D (25(OH)D) serves as a common marker for an individual’s vitamin D status.^[5]^ Several studies have hinted at a plausible link between diminished 25(OH)D levels and non-skeletal health issues, encompassing obesity,^[6]^ autoimmune diseases,^[7,8]^ cardiovascular diseases,^[9]^ diabetes,^[10]^ and cognitive impairments.^[11]^

The association between obesity and 25(OH)D remains a topic of debate. Pereira-Santos et al^[6]^ conducted a meta-analysis, revealing a 35% higher prevalence of vitamin D deficiency in obese individuals compared to those with normal weight. However, a cross-sectional survey of 1277 participants^[12]^ indicated, through multifactorial regression analysis, a lack of significant correlation between 25(OH)D levels and body mass index (BMI). Nevertheless, it highlighted a noteworthy negative correlation with waist-to-hip ratio (WHR) and body fat percentage (BFP). Mora et al,^[13]^ through a meta-analysis of randomized controlled trials, demonstrated that supplementing 25(OH)D did not significantly impact BMI. Mendelian randomization (MR), an epidemiological and genetic method, explores causal associations between exposure and outcomes. MR study results remain uninfluenced by confounding factors due to the random distribution of allele genes during gamete formation, ensuring higher quality outcomes.^[14]^ In line with this rationale, our study utilizes bidirectional Mendelian randomization analysis to comprehensively investigate the causal links between BMI, WHR, BFP, whole body fat mass (WFM), and 25(OH)D levels. This endeavor seeks to offer valuable insights into the prevention and management of both obesity and vitamin D deficiency.

## 2. Materials and methods

### 2.1. Research design

**Figure 1.**
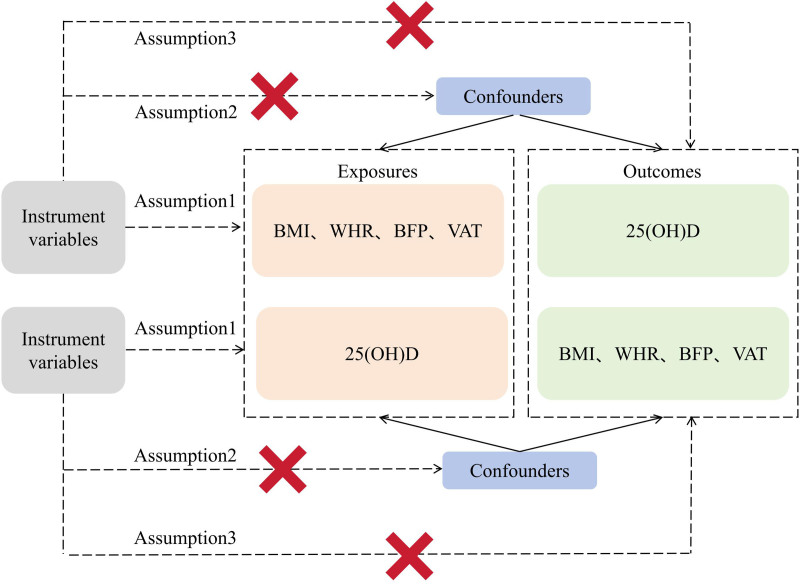
Diagram of the design idea of this study. BFP = body fat percentage, BMI = body mass index, VAT, WHR = waist-to-hip ratio.

This study utilizes BMI, WHR, BFP, and WFM as exposure factors, with 25(OH)D as the outcome, employing instrumental variable selection for forward MR analysis. To evaluate the study’s consistency, Cochran’s *Q* test is employed, followed by sensitivity analyses encompassing single-nucleotide polymorphism (SNP) effect analysis, horizontal pleiotropy analysis, and leave-one-out analysis to verify the reliability of the results. Furthermore, the study conducts reverse MR analysis, employing 25(OH)D as the exposure and BMI, WHR, BFP, and WFM as outcomes to explore reverse associations. Adherence to 3 core assumptions in MR studies is crucial: strong correlation between instrumental variables and exposure; independence of instrumental variables from confounding factors related to both exposure and outcome; sole impact of instrumental variables on the outcome through the exposure. Figure 1 illustrates the schematic representation of this study’s design rationale.

### 2.2. Data sources

Four indicators of obesity body composition were used in this study, including SNP data for BMI from a meta-analysis of 125 studies with a sample size of 339,224 cases by Locke et al.^[15]^ Instrumental variables for WHR were derived from a meta-analysis with a sample size of 224,459 cases from the largest consortium of genetic investigations of anthropometric traits (GIANT) in the United States.^[16]^ Instrumental variables for both BFP and WFM were obtained from pooled data from IEU open Genome-Wide Association Study, containing sample sizes of 454,633 and 454,137 cases, respectively. Genome-wide data on 25(OH)D were obtained from a Genome-Wide Association Study containing 417,580 individuals of European ancestry from the UK Biobank dataset^[17]^ (Table 1).

**Table 1:** Basic characteristics of the included data.

Type	Phenotype	SNP	Simple size	PubMed ID/IEU Open GWAS ID
Body composition	BMI	2555511	681,275	25673413
	WHR	2562516	224,459	25673412
	BFP	9851867	454,633	ukb-b-8909
	WFM	9851867	454,137	ukb-b-19393
25(OH)D	25(OH)D	8644129	417,580	32242144

BFP = body fat percentage, BMI = body mass index, GWAS = Genome-Wide Association Study, SNP = single-nucleotide polymorphism, WFM = whole body fat mass, WHR = waist-to-hip ratio.

### 2.3. Screening of instrumental variables

Since SNPs for MR studies must be closely related to exposure, SNPs were screened in this study with *P* < 5 × 10^−8^ as a threshold, and to prevent the occurrence of linkage disequilibrium, the parameter r^2^ < 0.001 was set, and SNPs with a distance of 10,000 kilobase pairs were analyzed. In addition, to prevent the weak instrumental variables from biasing the outcome, we evaluated the completed SNPs for screening based on the *F* statistic, which was calculated as^[18]^:


$$\begin{array}{l} F\;statistic=\frac{R^{2}(n-1-k)}{(1-R^{2})k} \end{array}$$

$$\begin{array}{l} R^{2}=\frac{2\times MAF\left(1-MAF\right)\times \beta^{2}}{SE^{2}\times n} \end{array}$$

In the formula, MAF = minor allele frequency, β = effect value, SE = standard error, n = sample size, and *k* = number of instrumental variables. It is usually considered that an instrumental variable is weakly instrumental if *F* statistic < 10, so this study was screened with a threshold of *F* > 10.^[19]^

### 2.4. Statistical analysis

This study employed 2-way Mendelian randomization analyses using the TwoSampleMR software package in R 4.2.3. Various methods, including inverse variance weighted (IVW), MR Egger regression, weighted median, weighted mode, and simple mode, were utilized to investigate the causal associations between the 4 body components and 25(OH)D levels. The initial analysis consisted of IVW, a fundamental method in Mendelian randomization (MR) analysis. IVW relies on the assumption that each SNP functions as a valid instrumental variable, enabling a dependable assessment of the causal relationship between exposure and outcome.^[20]^ MR Egger regression analysis was employed to deduce causal inference, particularly useful in scenarios involving potential pleiotropy or a substantial number of invalid instrumental variables.^[21]^ The weighted median approach was applied under the assumption of a minimum of 50% valid instrumental variables.^[22]^ Additionally, weighted mode and simple mode, while relaxing certain assumptions, demonstrate reduced test efficacy compared to the preceding 3 methods, serving as supplementary tools in MR analysis.^[20]^

### 2.5. Sensitivity analysis

In this study, rigorous sensitivity analyses were conducted using the TwoSampleMR and MR-PRESSO packages in R 4.2.3 to ensure the reliability of the findings. Firstly, Cochran’s *Q* test assessed SNP heterogeneity. Secondly, MR-Egger’s intercept test detected potential pleiotropy among the SNPs. Thirdly, MR-PRESSO identified and excluded significant outliers in the study results; subsequent analyses were conducted after their removal. Lastly, individual SNP effect analyses and sensitivity analyses using the leave-one-out method were employed to identify SNPs prone to significant heterogeneity.

## 3. Results

### 3.1. Causal effects of body composition on 25(OH)D

**Figure 2.**
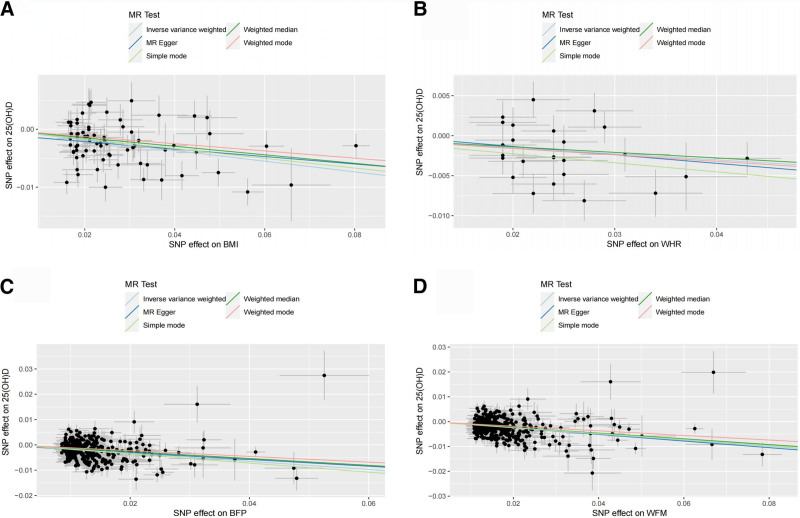
Scatter plot of causal effect of body composition on 25(OH)D. (A) Causal effect of BMI on 25(OH)D; (B) causal effect of WHR on 25(OH)D; (C) causal effect of BFP on 25(OH)D; (D) causal effect of WFM on 25(OH)D. BFP = body fat percentage, BMI = body mass index, MR = Mendelian randomization, SNP = single-nucleotide polymorphism, WFM = whole body fat mass, WHR = waist-to-hip ratio.

Following rigorous screening, MR analyses were conducted involving BMI, WHR, BFP, and WFM concerning 25(OH)D. Ultimately, 74, 25, 345, and 390 SNPs were included, respectively. In our study, the *F* statistic for each SNP was >10, indicating the absence of weak instrumental variables (Table S1, Supplemental Digital Content, http://links.lww.com/MD/O82). The findings indicated a significant causal relationship between all 4 body components investigated in this study and decreased 25(OH)D levels: BMI (IVW:OR = 0.912, 95% CI: 0.888–0.937, *P* < .001), WHR (IVW:OR = 0.927, 95% CI: 0.882–0.975, *P* = .003), BFP (IVW:OR = 0.883, 95% CI: 0.867–0.899, *P* < .001), WFM (IVW:OR = 0.850, 95% CI: 0.829–0.872, *P* < .001; Table 2; Fig. 2).

**Table 2:** Causal effects of body composition on 25(OH)D.

Exposure	Outcome	Methods	SNP	β	SE	*P*	OR(95%CI)
BMI	25(OH)D	MR Egger	74	−0.064	0.033	.055	0.938 (0.879, 1.000)
		Weighted median	74	−0.074	0.016	<.001	0.929 (0.901, 0.959)
		IVW	74	−0.092	0.014	<.001	0.912 (0.888, 0.937)
		Simple mode	74	−0.084	0.040	.038	0.920 (0.851, 0.994)
		Weighted mode	74	−0.062	0.023	.008	0.940 (0.898, 0.983)
WHR	25(OH)D	MR Egger	25	−0.106	0.110	.349	0.900 (0.725, 1.117)
		Weighted median	25	−0.070	0.029	.016	0.933 (0.881, 0.987)
		IVW	25	−0.076	0.026	.003	0.927 (0.882, 0.975)
		Simple mode	25	−0.113	0.060	.070	0.893 (0.795, 1.004)
		Weighted mode	25	−0.080	0.042	.069	0.923 (0.850, 1.002)
BFP	25(OH)D	MR Egger	345	−0.134	0.041	.001	0.875 (0.834, 0.919)
		Weighted median	345	−0.135	0.015	<.001	0.891 (0.872, 0.911)
		IVW	345	−0.162	0.013	<.001	0.883 (0.867, 0.899)
		Simple mode	345	−0.180	0.053	.001	0.887 (0.813, 0.968)
		Weighted mode	345	−0.114	0.036	.002	0.912 (0.860, 0.966)
WFM	25(OH)D	MR Egger	390	−0.133	0.025	<.001	0.875 (0.807, 0.948)
		Weighted median	390	−0.115	0.011	<.001	0.874 (0.848, 0.901)
		IVW	390	−0.125	0.009	<.001	0.850 (0.829, 0.872)
		Simple mode	390	−0.120	0.045	.008	0.836 (0.753, 0.927)
		Weighted mode	390	−0.093	0.030	.002	0.893 (0.832, 0.958)

BFP = body fat percentage, BMI = body mass index, IVW = inverse variance weighted, MR = Mendelian randomization, SNP = single-nucleotide polymorphism, WFM = whole body fat mass, WHR = waist-to-hip ratio.

### 3.2. Causal effects of 25(OH)D on body composition

**Figure 3.**
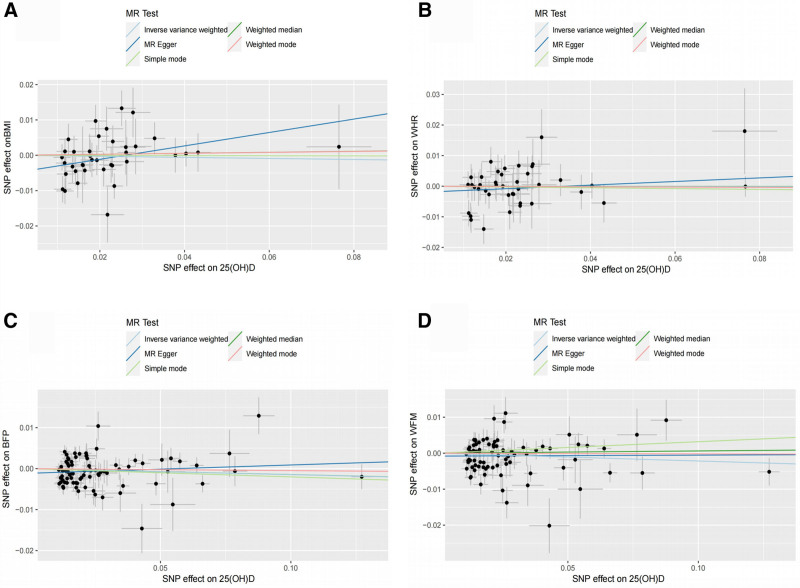
Scatter plot of causal effect of 25(OH)D on body composition. (A) Causal effect of 25(OH)D on BMI; (B) causal effect of 25(OH)D on WHR; (C) causal effect of 25(OH)D on BFP; (D) causal effect of 25(OH)D on WFM. BFP = body fat percentage, BMI = body mass index, MR = Mendelian randomization, SNP = single-nucleotide polymorphism, WFM = whole body fat mass, WHR = waist-to-hip ratio.

Following the screening process, 38, 41, 81, and 78 SNPs were included in the MR analysis correlating 25(OH)D with BMI, WHR, BFP, and WFM, respectively. In our study, the *F* statistic for each SNP was >10, indicating the absence of weak instrumental variables (Table S1, Supplemental Digital Content, http://links.lww.com/MD/O82). The outcomes indicated a lack of statistically significant causal relationships between 25(OH)D levels and any of the 4 body constituents: BMI (IVW: OR = 0.985, 95% CI: 0.90–91.067, *P* = .716), WHR (IVW:OR = 0.998, 95% CI: 0.939–1.061, *P* = .948), BFP (IVW: OR = 0.985, 95% CI: 0.962–1.009, *P* = .229), WFM (IVW:OR = 0.978, 95% CI: 0.949–1.009, and *P* = .170; Table 3; Fig. 3).

**Table 3:** Causal effects of 25(OH)D on body composition.

Exposure	Outcome	Methods	SNP	β	SE	*P*	OR(95%CI)
25(OH)D	BMI	MR Egger	38	0.190	0.097	.058	1.209 (1.000, 1.461)
		Weighted median	38	0.014	0.051	.786	1.014 (0.917, 1.122)
		IVW	38	−0.015	0.041	.716	0.985 (0.909, 1.067)
		Simple mode	38	−0.002	0.080	.981	0.998 (0.853, 1.167)
		Weighted mode	38	0.014	0.055	.808	1.014 (0.910, 1.130)
25(OH)D	WHR	MR Egger	41	0.059	0.057	.308	1.061 (0.949, 1.186)
		Weighted median	41	−0.001	0.039	.979	0.999 (0.926, 1.078)
		IVW	41	−0.002	0.031	.948	0.998 (0.939, 1.061)
		Simple mode	41	−0.013	0.083	.875	0.987 (0.839, 1.161)
		Weighted mode	41	−0.005	0.040	.906	0.995 (0.920, 1.077)
25(OH)D	BFP	MR Egger	81	0.020	0.021	.344	1.020 (0.979, 1.064)
		Weighted median	81	−0.014	0.014	.302	0.986 (0.959, 1.013)
		IVW	81	−0.015	0.012	.229	0.985 (0.962, 1.009)
		Simple mode	81	−0.020	0.027	.450	0.980 (0.930, 1.032)
		Weighted mode	81	−0.005	0.015	.751	0.995 (0.966, 1.025)
25(OH)D	WFM	MR Egger	78	0.003	0.028	.923	1.003 (0.950, 1.059)
		Weighted median	78	0.006	0.018	.734	1.006 (0.971, 1.043)
		IVW	78	−0.022	0.016	.170	0.978 (0.949, 1.009)
		Simple mode	78	0.032	0.036	.383	1.032 (0.961, 1.109)
		Weighted mode	78	−0.002	0.018	.922	0.998 (0.964, 1.033)

BFP = body fat percentage, BMI = body mass index, IVW = inverse variance weighted, MR = Mendelian randomization, SNP = single-nucleotide polymorphism, WFM = whole body fat mass, WHR = waist-to-hip ratio.

### 3.3. Sensitivity analysis for 2-way MR analysis

**Figure 4.**
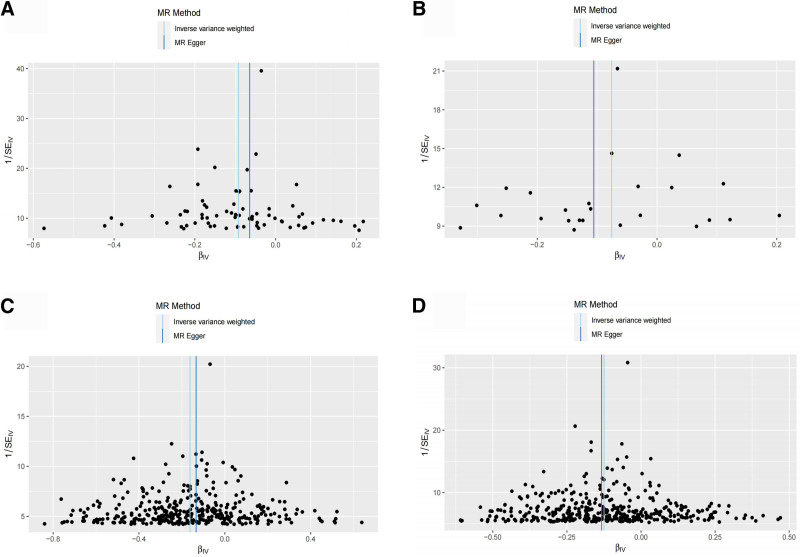
Funnel plot of MR analysis of 25(OH)D by human body composition. (A) Funnel plot of BMI on 25(OH)D; (B) funnel plot of WHR on 25(OH)D; (C) funnel plot of BFP on 25(OH)D; (D) funnel plot of WFM on 25(OH)D. BFP = body fat percentage, BMI = body mass index, MR = Mendelian randomization, WFM = whole body fat mass, WHR = waist-to-hip ratio.

**Figure 5.**
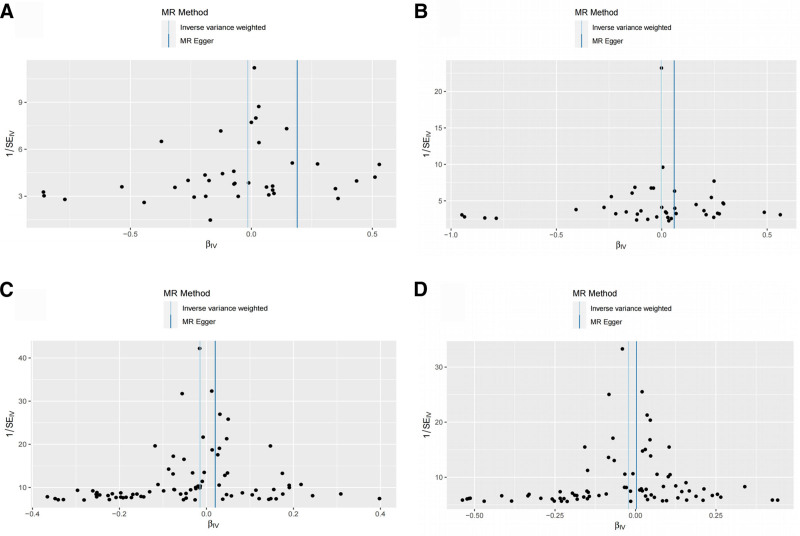
Funnel plot of MR analysis of body composition by 25(OH)D. (A) Funnel plot of 25(OH)D against BMI; (B) funnel plot of 25(OH)D against WHR; (C) funnel plot of 25(OH)D against BFP; (D) funnel plot of 25(OH)D against WFM. BFP = body fat percentage, BMI = body mass index, MR = Mendelian randomization, WFM = whole body fat mass, WHR = waist-to-hip ratio.

The Cochran’s *Q* test within the IVW results indicated no significant heterogeneity in this study (*P* > .05). Similarly, the MR-Egger intercept test exhibited no significant horizontal pleiotropy (*P* > .05), as depicted in Table 4. Sensitivity analysis, employing the leave-one-out method and assessing individual SNP effects, revealed no SNPs with substantial influence on the overall study effect. Additionally, the funnel plot illustrated a symmetric scattering of instrumental variables, suggesting minimal heterogeneity (Figs. 4 and 5). In conclusion, this 2-way MR study showcases relatively robust results.

**Table 4:** Results of Cochran’s *Q* test and multiple validity tests.

Exposure	Outcome	*Q* statistics (*P*)	Pleiotropic test (*P*)
BMI	25(OH)D	.149	.358
WHR	25(OH)D	.170	.783
BFP	25(OH)D	.434	.463
WFM	25(OH)D	.350	.720
25(OH)D	BMI	.170	.268
25(OH)D	WHR	.106	.209
25(OH)D	BFP	.166	.493
25(OH)D	WFM	.143	.288

BFP = body fat percentage, BMI = body mass index, WFM = whole body fat mass, WHR = waist-to-hip ratio.

## 4. Discussion

Numerous previous observational studies and meta-analyses have indicated a robust correlation between obesity and diminished vitamin D levels.^[6]^ Further research has revealed that vitamin D deficiency elevates parathyroid hormone levels, potentially enhancing calcium ion influx into adipocytes,^[23]^. Simultaneously, it expedites the differentiation of preadipocytes into adipocytes,^[24]^ thereby potentially contributing directly or indirectly to an increased risk of obesity. This intricate relationship between obesity and vitamin D levels underscores its complexity, where the causal association remains partly contentious. Elucidating this causal link can offer valuable insights into combating both obesity and vitamin D deficiency.

This study pioneers the application of 2-way MR analysis to explore causal relationships between 4 obesity-related body components, BMI, WHR, BFP, and WFM, and 25(OH)D levels. Positive MR outcomes confirm significant causal links between all 4 obesity-related body components and reduced 25(OH)D levels. The most pronounced negative causal association appears between WFM and 25(OH)D levels (IVW: OR = 0.850, 95% CI: 0.829–0.872, *P* < .001), followed by BFP(IVW: OR = 0.883, 95% CI: 0.867–0.899, *P* < .001), aligning with earlier observational findings. Previous studies indicated that reduced outdoor physical activity in obese individuals correlates with lower 25(OH)D levels.^[25]^ Sunlight’s ultraviolet radiation promotes the conversion of 7-deoxycholesterol to vitamin D3 precursors in the skin, augmenting 25(OH)D levels.^[4]^ The limited outdoor activity in obese patients hampers this process. Research suggests that the fat-soluble nature of vitamin D may be sequestered in the fat depots of obese patients, reducing its bioavailability,^[26]^ further supporting the positive causal link between obesity and low 25(OH)D levels. Conversely, inverse MR analysis unveils no significant causal relationship between 25(OH)D levels and the 4 body compositions, consistent with meta-analyses demonstrating no notable effect of vitamin D supplementation on obesity.^[27]^ Despite the absence of a significant causal link between 25(OH)D levels and the 4 body compositions in this study, their directionality leans toward a negative correlation: i.e., all OR values were < 0. An experimental model inducing obesity in mice through a high-fat diet demonstrated that elevated vitamin D and calcium intake could activate the apoptotic pathway in adipose tissue calcium ion streets, potentially reducing adipocytes and mitigating obesity.^[28]^ However, this association is yet to be validated in humans. When considered alongside this study’s outcomes, the hypothesis of improving obesity through vitamin D supplementation lacks substantiation.

This study represents the inaugural bivariate causal association exploration employing bidirectional MR analysis to elucidate the genetic underpinnings of the relationship between obesity and 25(OH)D levels. This method mitigates confounding factors present in traditional observational studies, providing a more precise causal inference. Nevertheless, certain limitations merit acknowledgment: The majority of genetic instrumental variables were derived from European populations, possibly introducing bias due to ethnic influences. The study did not differentiate between various adipose tissues (e.g., visceral fat, lower limb fat), resulting in an imprecise assessment of causality. Future research endeavors should expand sample sizes, employ stratification, include diverse subgroups, and delve deeper into the intrinsic mechanisms governing this causal association. To summarize, this study delineated a substantial causal relationship between 4 obesity-related body components (BMI, WHR, BFP, and WFM) and decreased 25(OH)D levels, with BFP exhibiting the most pronounced effect. However, no evidence supports reverse causality between obesity and 25(OH)D levels.

## Acknowledgments

We expressed our sincere thanks to all the patients who participated in this study.

## Author contributions

**Conceptualization:** Li Jixin, Wenru Wang.

**Data curation:** Li Jixin, Wenru Wang.

**Formal analysis:** Li Jixin, Wenru Wang.

**Funding acquisition:** Jin Zhang.

**Investigation:** Linjie Qiu.

**Methodology:** Wenru Wang, Linjie Qiu, Yan Ren.

**Resources:** Li Jixin.

**Software:** Wenru Wang, Yan Ren.

**Supervision:** Linjie Qiu.

**Validation:** Meijie Li, Wenjie Li.

**Writing – original draft:** Li Jixin.

**Writing – review & editing:** Li Jixin, Wenru Wang.

## Supplementary Material


