# Supplementary material for: The causal association between body composition and 25-hydroxyvitamin D levels: A bidirectional Mendelian randomization analysis
Source: Medicine (Baltimore). 2024 Dec 13;103(50):e40618. doi: 10.1097/MD.0000000000040618 (PMC11651481; doi:10.1097/MD.0000000000040618)
Supplement: Supplementary file 1 [file medi-103-e40618-s001.docx]

**Supplementary table1 Basic characteristics of SNPs**

| exposure | beta | se | pval | SNP | effect_allele | other_allele | eaf | F-statistic |
| --- | --- | --- | --- | --- | --- | --- | --- | --- |
| 25(OH)D | -0.0147881 | 0.00201077 | 1.92E-13 | rs6671730 | A | G | 0.434286 | 54.08788048 |
| 25(OH)D | 0.0164389 | 0.00224585 | 2.49E-13 | rs10908469 | C | A | 0.26971 | 53.57769361 |
| 25(OH)D | -0.0134641 | 0.00202533 | 2.97E-11 | rs7522116 | T | C | 0.566233 | 44.193975 |
| 25(OH)D | 0.0197401 | 0.00238729 | 1.35E-16 | rs7528419 | G | A | 0.224671 | 68.37358302 |
| 25(OH)D | -0.0214952 | 0.00209979 | 1.36E-24 | rs35408430 | T | C | 0.342194 | 104.7927541 |
| 25(OH)D | 0.0876986 | 0.00595617 | 4.52E-49 | rs61816766 | C | T | 0.029104 | 216.7959432 |
| 25(OH)D | 0.0175857 | 0.00250898 | 2.40E-12 | rs6672758 | T | C | 0.800872 | 49.12752908 |
| 25(OH)D | -0.0229402 | 0.0020845 | 3.61E-28 | rs2131925 | T | G | 0.643625 | 121.1129551 |
| 25(OH)D | 0.0785529 | 0.00467652 | 2.55E-63 | rs12123821 | T | C | 0.047527 | 282.1492198 |
| 25(OH)D | -0.0144848 | 0.00214513 | 1.45E-11 | rs2642439 | G | A | 0.685074 | 45.59505804 |
| 25(OH)D | -0.0483073 | 0.00366555 | 1.16E-39 | rs2012736 | A | C | 0.080814 | 173.6789693 |
| 25(OH)D | -0.0116434 | 0.00202406 | 8.79E-09 | rs58235267 | G | C | 0.487526 | 33.09122698 |
| 25(OH)D | -0.0152142 | 0.00214041 | 1.18E-12 | rs1047891 | A | C | 0.315821 | 50.52476467 |
| 25(OH)D | 0.0206128 | 0.00203644 | 4.42E-24 | rs1260326 | C | T | 0.606565 | 102.4544299 |
| 25(OH)D | -0.0140184 | 0.00206152 | 1.05E-11 | rs727857 | A | G | 0.611489 | 46.24042221 |
| 25(OH)D | -0.0161021 | 0.00261564 | 7.46E-10 | rs3849374 | C | G | 0.178029 | 37.89737232 |
| 25(OH)D | 0.01425 | 0.00221206 | 1.18E-10 | rs7569755 | A | G | 0.29058 | 41.49883677 |
| 25(OH)D | 0.0261229 | 0.00459423 | 1.30E-08 | rs6547409 | T | C | 0.049783 | 32.33086388 |
| 25(OH)D | -0.0172156 | 0.00208415 | 1.45E-16 | rs6782190 | A | G | 0.647512 | 68.23172977 |
| 25(OH)D | -0.0124079 | 0.0022155 | 2.14E-08 | rs10642047 | ATTTC | A | 0.71562 | 31.36556107 |
| 25(OH)D | 0.0140213 | 0.00225294 | 4.86E-10 | rs9861009 | C | T | 0.727515 | 38.73265893 |
| 25(OH)D | 0.0138401 | 0.00226747 | 1.04E-09 | rs11458206 | TG | T | 0.272091 | 37.25592551 |
| 25(OH)D | 0.0136408 | 0.00201094 | 1.17E-11 | rs11721204 | T | C | 0.438074 | 46.01309614 |
| 25(OH)D | 0.05745 | 0.00276634 | 8.51E-96 | rs28437159 | T | C | 0.154162 | 431.2895675 |
| 25(OH)D | -0.054035 | 0.0032715 | 2.78E-61 | rs113209890 | T | C | 0.102968 | 272.8075984 |
| 25(OH)D | -0.0211949 | 0.00325203 | 7.15E-11 | rs78649910 | A | T | 0.106179 | 42.47698246 |
| 25(OH)D | 0.0159119 | 0.00250595 | 2.16E-10 | rs4364259 | A | G | 0.202148 | 40.31802767 |
| 25(OH)D | -0.0160047 | 0.00217341 | 1.79E-13 | rs11732896 | A | G | 0.298791 | 54.22652245 |
| 25(OH)D | 0.0528039 | 0.00671977 | 3.90E-15 | rs189407772 | G | A | 0.022694 | 61.74804084 |
| 25(OH)D | -0.0270859 | 0.0026234 | 5.45E-25 | rs140589749 | T | G | 0.174322 | 106.6000757 |
| 25(OH)D | 0.0294988 | 0.00402809 | 2.42E-13 | rs13137398 | T | A | 0.065246 | 53.63031747 |
| 25(OH)D | 0.0784564 | 0.00210611 | 1.00E-200 | rs6837680 | T | A | 0.656939 | 1387.69701 |
| 25(OH)D | -0.0118157 | 0.00215476 | 4.17E-08 | rs113529602 | C | T | 0.309154 | 30.06914654 |
| 25(OH)D | -0.012286 | 0.00201755 | 1.13E-09 | rs4616820 | T | C | 0.464954 | 37.08279063 |
| 25(OH)D | -0.0639057 | 0.0025722 | 2.94E-136 | rs35846253 | T | C | 0.186629 | 617.2622603 |
| 25(OH)D | 0.0132137 | 0.00219753 | 1.82E-09 | rs10070734 | C | T | 0.709531 | 36.15590692 |
| 25(OH)D | -0.014528 | 0.00264902 | 4.15E-08 | rs31612 | C | T | 0.174438 | 30.07746208 |
| 25(OH)D | 0.0112045 | 0.00202666 | 3.23E-08 | rs9325107 | T | G | 0.441866 | 30.56491342 |
| 25(OH)D | -0.0135623 | 0.00232502 | 5.44E-09 | rs28374650 | T | C | 0.243562 | 34.0261888 |
| 25(OH)D | 0.011051 | 0.00201182 | 3.95E-08 | rs9490317 | C | T | 0.445894 | 30.17344621 |
| 25(OH)D | -0.0233623 | 0.0026823 | 3.04E-18 | rs2248551 | A | G | 0.165222 | 75.86063851 |
| 25(OH)D | -0.0249871 | 0.00385103 | 8.67E-11 | rs72834856 | G | T | 0.072064 | 42.09959385 |
| 25(OH)D | 0.0117571 | 0.00200093 | 4.21E-09 | rs9476310 | T | C | 0.511363 | 34.52523417 |
| 25(OH)D | -0.0166065 | 0.00282521 | 4.15E-09 | rs75741381 | G | C | 0.147638 | 34.55053289 |
| 25(OH)D | -0.0138826 | 0.00243596 | 1.20E-08 | rs2346264 | C | A | 0.782685 | 32.47890027 |
| 25(OH)D | -0.0145575 | 0.00223829 | 7.83E-11 | rs10085881 | C | T | 0.282185 | 42.30005078 |
| 25(OH)D | -0.0117532 | 0.00203758 | 8.01E-09 | rs6966728 | T | C | 0.462632 | 33.27230701 |
| 25(OH)D | 0.0138131 | 0.00207209 | 2.62E-11 | rs7784802 | T | A | 0.360991 | 44.43908233 |
| 25(OH)D | 0.0132996 | 0.00202139 | 4.72E-11 | rs804281 | G | A | 0.583605 | 43.28893813 |
| 25(OH)D | -0.0176145 | 0.00294133 | 2.12E-09 | rs57459725 | G | C | 0.1329 | 35.86353829 |
| 25(OH)D | -0.0234029 | 0.00202418 | 6.44E-31 | rs12056768 | G | T | 0.582909 | 133.6721997 |
| 25(OH)D | 0.0148311 | 0.00230018 | 1.13E-10 | rs28692966 | A | G | 0.252936 | 41.57412145 |
| 25(OH)D | -0.0185345 | 0.00256813 | 5.31E-13 | rs532436 | A | G | 0.183792 | 52.08680769 |
| 25(OH)D | 0.0175688 | 0.00313411 | 2.07E-08 | rs13284054 | C | T | 0.117727 | 31.42358479 |
| 25(OH)D | -0.019633 | 0.00288097 | 9.44E-12 | rs9409266 | A | G | 0.861101 | 46.44039033 |
| 25(OH)D | 0.0152166 | 0.00249607 | 1.09E-09 | rs3925446 | A | G | 0.199129 | 37.16393807 |
| 25(OH)D | 0.0265692 | 0.00440069 | 1.56E-09 | rs77532868 | T | C | 0.054042 | 36.45149989 |
| 25(OH)D | 0.0215803 | 0.00387022 | 2.46E-08 | rs11022863 | T | C | 0.071883 | 31.09164138 |
| 25(OH)D | -0.127167 | 0.00395711 | 1.00E-200 | rs61147618 | T | C | 0.069062 | 1032.743823 |
| 25(OH)D | -0.0766273 | 0.00199958 | 1.00E-200 | rs10766197 | A | G | 0.456295 | 1468.552504 |
| 25(OH)D | -0.0986737 | 0.00257961 | 1.00E-200 | rs10898144 | T | A | 0.182852 | 1463.169781 |
| 25(OH)D | 0.0189846 | 0.00245472 | 1.04E-14 | rs1872285 | A | G | 0.206423 | 59.81346751 |
| 25(OH)D | -0.0547354 | 0.00863527 | 2.32E-10 | rs117862422 | C | T | 0.013593 | 40.17767024 |
| 25(OH)D | -0.0346049 | 0.00600757 | 8.40E-09 | rs71467497 | C | T | 0.028603 | 33.18008677 |
| 25(OH)D | 0.0431755 | 0.00294423 | 1.09E-48 | rs964184 | C | G | 0.868376 | 215.0459581 |
| 25(OH)D | -0.0505488 | 0.00518915 | 2.01E-22 | rs72858657 | C | T | 0.038801 | 94.89192364 |
| 25(OH)D | 0.0116703 | 0.00206786 | 1.66E-08 | rs6591216 | T | G | 0.390939 | 31.85090492 |
| 25(OH)D | -0.076465 | 0.00762946 | 1.22E-23 | rs7112442 | T | C | 0.01741 | 100.4471884 |
| 25(OH)D | -0.021925 | 0.00302751 | 4.42E-13 | rs2847500 | A | G | 0.123503 | 52.445476 |
| 25(OH)D | 0.0120567 | 0.002018 | 2.31E-09 | rs1038165 | T | C | 0.583349 | 35.69559173 |
| 25(OH)D | -0.0217954 | 0.00387948 | 1.93E-08 | rs12372115 | T | G | 0.070719 | 31.56331816 |
| 25(OH)D | -0.0125352 | 0.00199379 | 3.23E-10 | rs11182428 | C | T | 0.519995 | 39.52789691 |
| 25(OH)D | -0.0403465 | 0.0020206 | 1.06E-88 | rs10859995 | C | T | 0.582634 | 398.7044062 |
| 25(OH)D | 0.0216996 | 0.00382584 | 1.41E-08 | rs73413596 | C | T | 0.073854 | 32.16991028 |
| 25(OH)D | -0.0208967 | 0.00278474 | 6.19E-14 | rs12317268 | G | A | 0.151004 | 56.3100755 |
| 25(OH)D | -0.0126503 | 0.00220304 | 9.35E-09 | rs9569209 | T | C | 0.285576 | 32.97288019 |
| 25(OH)D | -0.0378247 | 0.00260904 | 1.26E-47 | rs8018720 | C | G | 0.823327 | 210.179091 |
| 25(OH)D | -0.0128133 | 0.00210896 | 1.23E-09 | rs4900599 | T | G | 0.338865 | 36.91350665 |
| 25(OH)D | 0.0129076 | 0.00208651 | 6.16E-10 | rs35831632 | AT | A | 0.35594 | 38.26925923 |
| 25(OH)D | -0.0141728 | 0.00221789 | 1.66E-10 | rs325384 | T | C | 0.284205 | 40.83488263 |
| 25(OH)D | -0.0329215 | 0.00242187 | 4.38E-42 | rs1800588 | T | C | 0.215203 | 184.7811111 |
| 25(OH)D | -0.0158394 | 0.00261723 | 1.43E-09 | rs62012775 | T | A | 0.175942 | 36.62635271 |
| 25(OH)D | 0.0261937 | 0.00204572 | 1.55E-37 | rs1532085 | G | A | 0.614835 | 163.9461854 |
| 25(OH)D | -0.0133407 | 0.00219977 | 1.32E-09 | rs62007299 | A | G | 0.712537 | 36.77923453 |
| 25(OH)D | -0.0166321 | 0.00255158 | 7.11E-11 | rs77924615 | A | G | 0.193485 | 42.48893019 |
| 25(OH)D | 0.0147611 | 0.00225195 | 5.57E-11 | rs8063565 | C | G | 0.733582 | 42.96550864 |
| 25(OH)D | 0.0230493 | 0.00260705 | 9.48E-19 | rs11076175 | G | A | 0.178358 | 78.16579694 |
| 25(OH)D | -0.0243589 | 0.00439221 | 2.92E-08 | rs4327060 | T | C | 0.054396 | 30.75736683 |
| 25(OH)D | 0.0137025 | 0.00225257 | 1.18E-09 | rs138385079 | TG | T | 0.273173 | 37.00351924 |
| 25(OH)D | 0.0428822 | 0.00774142 | 3.04E-08 | rs139861017 | G | C | 0.016773 | 30.68406229 |
| 25(OH)D | -0.023334 | 0.00291776 | 1.27E-15 | rs11542462 | A | G | 0.134344 | 63.95569971 |
| 25(OH)D | -0.0135306 | 0.00220667 | 8.70E-10 | rs10454087 | T | C | 0.284822 | 37.59753127 |
| 25(OH)D | 0.017715 | 0.00249217 | 1.18E-12 | rs2952289 | T | C | 0.798032 | 50.52740401 |
| 25(OH)D | -0.0263626 | 0.0040284 | 5.98E-11 | rs8091117 | A | C | 0.065298 | 42.82637403 |
| 25(OH)D | -0.0192879 | 0.00277797 | 3.83E-12 | rs4121823 | A | T | 0.845333 | 48.20751985 |
| 25(OH)D | 0.0181228 | 0.00267963 | 1.35E-11 | rs2037511 | A | G | 0.166007 | 45.74050264 |
| 25(OH)D | 0.0407994 | 0.00383081 | 1.74E-26 | rs200210321 | AG | A | 0.073153 | 113.429581 |
| 25(OH)D | -0.0123751 | 0.00208027 | 2.70E-09 | rs12462826 | A | G | 0.368722 | 35.38816324 |
| 25(OH)D | -0.012558 | 0.00214992 | 5.18E-09 | rs3814995 | T | C | 0.311595 | 34.11900502 |
| 25(OH)D | -0.0163068 | 0.00255729 | 1.81E-10 | rs12721051 | G | C | 0.186482 | 40.66095069 |
| 25(OH)D | 0.0255317 | 0.00314553 | 4.79E-16 | rs142158911 | A | G | 0.114608 | 65.88276271 |
| 25(OH)D | -0.0661522 | 0.00269018 | 1.60E-133 | rs212100 | C | T | 0.835999 | 604.680418 |
| 25(OH)D | 0.0284424 | 0.00366643 | 8.66E-15 | rs1048328 | A | G | 0.080247 | 60.1790994 |
| 25(OH)D | 0.0341831 | 0.00331429 | 6.10E-25 | rs6123359 | G | A | 0.102225 | 106.3755646 |
| 25(OH)D | -0.0226477 | 0.00278157 | 3.89E-16 | rs2616279 | T | C | 0.151761 | 66.29308347 |
| 25(OH)D | -0.0345955 | 0.00557773 | 5.56E-10 | rs2207132 | A | G | 0.03289 | 38.47018264 |
| 25(OH)D | 0.0356675 | 0.00237687 | 6.70E-51 | rs2585442 | G | C | 0.240654 | 225.1824197 |
| 25(OH)D | -0.0251483 | 0.00327069 | 1.48E-14 | rs2229742 | C | G | 0.103451 | 59.12055451 |
| 25(OH)D | -0.0124309 | 0.00212296 | 4.76E-09 | rs115621755 | T | C | 0.32712 | 34.2863712 |
| 25(OH)D | 0.0278196 | 0.00407045 | 8.23E-12 | rs2074735 | C | G | 0.064096 | 46.71075788 |
| 25(OH)D | -0.0119615 | 0.00212333 | 1.77E-08 | rs6003465 | C | T | 0.331994 | 31.73483887 |
| BFP | -0.0130789 | 0.00206635 | 2.50E-10 | rs10799778 | G | T | 0.833671 | 40.06218843 |
| BFP | 0.0474389 | 0.00485686 | 1.60E-22 | rs17024393 | C | T | 0.025909 | 95.40212543 |
| BFP | 0.018328 | 0.00156672 | 1.30E-31 | rs11205303 | C | T | 0.406646 | 136.8508116 |
| BFP | -0.0177569 | 0.00249206 | 1.00E-12 | rs35154152 | C | T | 0.106824 | 50.77118606 |
| BFP | -0.0322259 | 0.00466633 | 5.00E-12 | rs79518326 | A | C | 0.02864 | 47.69350205 |
| BFP | -0.00955748 | 0.00159404 | 2.00E-09 | rs9645335 | T | C | 0.367818 | 35.94912842 |
| BFP | 0.0127442 | 0.00178122 | 8.40E-13 | rs1377184 | T | A | 0.748963 | 51.19057775 |
| BFP | -0.0145771 | 0.00220294 | 3.70E-11 | rs12042959 | G | A | 0.143917 | 43.78616696 |
| BFP | 0.00966112 | 0.00154844 | 4.40E-10 | rs4908676 | G | A | 0.457718 | 38.92836733 |
| BFP | -0.0102079 | 0.00162025 | 3.00E-10 | rs3765971 | T | C | 0.65847 | 39.69252743 |
| BFP | -0.00950812 | 0.00169742 | 2.10E-08 | rs7535438 | A | C | 0.290883 | 31.37694659 |
| BFP | 0.0142768 | 0.00158851 | 2.50E-19 | rs6699744 | T | A | 0.616045 | 80.77590733 |
| BFP | -0.0146759 | 0.00190484 | 1.30E-14 | rs12724928 | C | T | 0.20486 | 59.35980739 |
| BFP | -0.00909072 | 0.00154941 | 4.40E-09 | rs815163 | C | T | 0.563216 | 34.42419979 |
| BFP | 0.0136291 | 0.00162387 | 4.70E-17 | rs2678204 | G | T | 0.340185 | 70.44202611 |
| BFP | 0.0157919 | 0.00237893 | 3.20E-11 | rs1318408 | G | A | 0.119506 | 44.06618407 |
| BFP | 0.0140143 | 0.00203896 | 6.30E-12 | rs3766823 | A | G | 0.172004 | 47.24168813 |
| BFP | -0.0108576 | 0.00192322 | 1.60E-08 | rs1284373 | T | C | 0.199214 | 31.87203125 |
| BFP | -0.0136932 | 0.0016614 | 1.70E-16 | rs6693294 | G | A | 0.688566 | 67.9299806 |
| BFP | -0.0139271 | 0.00155866 | 4.10E-19 | rs1013293 | A | G | 0.430286 | 79.83964688 |
| BFP | 0.0236276 | 0.00236585 | 1.70E-23 | rs71658797 | A | T | 0.120747 | 99.73895369 |
| BFP | 0.0105922 | 0.0016789 | 2.80E-10 | rs6688826 | C | T | 0.298206 | 39.80363056 |
| BFP | 0.0105954 | 0.00156929 | 1.50E-11 | rs2802774 | A | C | 0.547302 | 45.58566549 |
| BFP | 0.0134145 | 0.00184627 | 3.70E-13 | rs12072739 | G | A | 0.224584 | 52.79083773 |
| BFP | -0.0101759 | 0.00157772 | 1.10E-10 | rs12402939 | C | A | 0.392363 | 41.59927858 |
| BFP | 0.0210728 | 0.00168127 | 4.90E-36 | rs2785988 | A | C | 0.297382 | 157.0976047 |
| BFP | -0.0128866 | 0.00169042 | 2.50E-14 | rs72634814 | A | G | 0.345475 | 58.11489423 |
| BFP | 0.0158823 | 0.00259576 | 9.40E-10 | rs2640465 | G | A | 0.902017 | 37.43671375 |
| BFP | 0.0117369 | 0.0015617 | 5.70E-14 | rs2984618 | T | G | 0.416215 | 56.4821979 |
| BFP | 0.010317 | 0.00154445 | 2.40E-11 | rs11208779 | C | G | 0.528878 | 44.62304013 |
| BFP | 0.0146762 | 0.00156269 | 5.90E-21 | rs11165643 | T | C | 0.590144 | 88.20263852 |
| BFP | 0.0301643 | 0.00190436 | 1.70E-56 | rs543874 | G | A | 0.20527 | 250.8929172 |
| BFP | -0.00965759 | 0.00157945 | 9.70E-10 | rs11119208 | G | A | 0.614902 | 37.38744299 |
| BFP | -0.0101571 | 0.00157943 | 1.30E-10 | rs11122450 | G | T | 0.611689 | 41.35601563 |
| BFP | 0.0322372 | 0.00573378 | 1.90E-08 | rs76856798 | T | C | 0.018465 | 31.61058814 |
| BFP | 0.00961952 | 0.00175567 | 4.30E-08 | rs13026103 | A | G | 0.74098 | 30.02071471 |
| BFP | -0.0150933 | 0.00190234 | 2.10E-15 | rs1456014 | G | A | 0.205602 | 62.94947741 |
| BFP | -0.0130723 | 0.0015614 | 5.70E-17 | rs13408397 | T | C | 0.411055 | 70.09316361 |
| BFP | 0.0126967 | 0.00230925 | 3.80E-08 | rs6749911 | A | G | 0.126972 | 30.23011586 |
| BFP | -0.0111228 | 0.00155872 | 9.60E-13 | rs429343 | G | A | 0.576575 | 50.92042745 |
| BFP | 0.0126532 | 0.00173398 | 2.90E-13 | rs1453055 | A | G | 0.273355 | 53.24913747 |
| BFP | 0.0123638 | 0.0016188 | 2.20E-14 | rs10209821 | T | C | 0.342612 | 58.33343501 |
| BFP | -0.0479135 | 0.00359643 | 1.70E-40 | rs62107261 | C | T | 0.048327 | 177.4894606 |
| BFP | 0.028139 | 0.00203811 | 2.30E-43 | rs6744646 | G | A | 0.828299 | 190.6171971 |
| BFP | 0.0224027 | 0.00153822 | 4.80E-48 | rs6752378 | A | C | 0.486256 | 212.1113064 |
| BFP | -0.00988584 | 0.00154662 | 1.60E-10 | rs12475388 | A | G | 0.485685 | 40.85638249 |
| BFP | 0.0125399 | 0.00159364 | 3.60E-15 | rs10175266 | G | A | 0.368852 | 61.9166859 |
| BFP | -0.010248 | 0.00159844 | 1.40E-10 | rs10187101 | T | C | 0.363611 | 41.104139 |
| BFP | -0.0135852 | 0.0015523 | 2.10E-18 | rs1861410 | T | C | 0.555344 | 76.59153226 |
| BFP | -0.00986214 | 0.00170937 | 8.00E-09 | rs396354 | C | T | 0.715662 | 33.28665627 |
| BFP | 0.0119298 | 0.00154217 | 1.00E-14 | rs441792 | G | A | 0.486601 | 59.84141095 |
| BFP | 0.0170899 | 0.00157313 | 1.70E-27 | rs13389219 | T | C | 0.392441 | 118.0184255 |
| BFP | -0.00932639 | 0.00161898 | 8.40E-09 | rs6754292 | T | C | 0.644629 | 33.18517996 |
| BFP | -0.0153971 | 0.00157743 | 1.70E-22 | rs7575523 | G | T | 0.603242 | 95.27471726 |
| BFP | 0.00909731 | 0.0016107 | 1.60E-08 | rs2692741 | C | G | 0.367194 | 31.9004398 |
| BFP | -0.00923656 | 0.00154968 | 2.50E-09 | rs11677541 | A | G | 0.535271 | 35.52519375 |
| BFP | -0.0114802 | 0.00176393 | 7.60E-11 | rs3754963 | T | A | 0.255074 | 42.35807501 |
| BFP | 0.0085494 | 0.00156226 | 4.40E-08 | rs7598246 | C | T | 0.583694 | 29.9477809 |
| BFP | -0.0138669 | 0.0019837 | 2.70E-12 | rs72917533 | C | T | 0.185499 | 48.86599888 |
| BFP | -0.0117765 | 0.00192871 | 1.00E-09 | rs6750646 | T | C | 0.201154 | 37.28194903 |
| BFP | -0.0211076 | 0.00289361 | 3.00E-13 | rs4482463 | A | C | 0.923053 | 53.21054629 |
| BFP | 0.0103311 | 0.00171169 | 1.60E-09 | rs3791709 | T | A | 0.282417 | 36.42863546 |
| BFP | -0.0144544 | 0.0015682 | 3.00E-20 | rs12619178 | T | C | 0.400829 | 84.95662255 |
| BFP | 0.00856437 | 0.00154317 | 2.90E-08 | rs12053559 | G | T | 0.471201 | 30.80089239 |
| BFP | -0.00975461 | 0.00173683 | 2.00E-08 | rs56328878 | A | C | 0.267194 | 31.54315986 |
| BFP | 0.0151644 | 0.00165383 | 4.80E-20 | rs62190394 | T | C | 0.317033 | 84.07535927 |
| BFP | -0.0152608 | 0.00274222 | 2.60E-08 | rs2702123 | C | T | 0.086 | 30.97062813 |
| BFP | 0.00998353 | 0.00161504 | 6.30E-10 | rs2888778 | G | T | 0.652153 | 38.21216888 |
| BFP | -0.0163391 | 0.00274979 | 2.80E-09 | rs58120873 | A | G | 0.08686 | 35.30670631 |
| BFP | -0.0105978 | 0.00183563 | 7.80E-09 | rs4547574 | T | A | 0.227697 | 33.33198249 |
| BFP | -0.0115311 | 0.00158686 | 3.70E-13 | rs10496731 | G | T | 0.374905 | 52.80368734 |
| BFP | -0.0155134 | 0.00163892 | 2.90E-21 | rs2943653 | T | C | 0.672855 | 89.59803209 |
| BFP | 0.0301151 | 0.0023675 | 4.60E-37 | rs1801282 | G | C | 0.119535 | 161.8037728 |
| BFP | -0.0134993 | 0.00194516 | 3.90E-12 | rs33503 | A | G | 0.805882 | 48.16281223 |
| BFP | 0.0107963 | 0.00156132 | 4.70E-12 | rs1436348 | G | A | 0.582718 | 47.81520719 |
| BFP | 0.0096786 | 0.00170107 | 1.30E-08 | rs1964675 | T | C | 0.711087 | 32.3728334 |
| BFP | -0.0102573 | 0.00159162 | 1.20E-10 | rs262953 | A | G | 0.625978 | 41.53242982 |
| BFP | -0.00912091 | 0.00162066 | 1.80E-08 | rs2960420 | G | C | 0.350731 | 31.67324164 |
| BFP | -0.0143309 | 0.00216477 | 3.60E-11 | rs17639996 | A | G | 0.150071 | 43.82514984 |
| BFP | -0.0195859 | 0.00321452 | 1.10E-09 | rs114295766 | T | A | 0.069527 | 37.12400318 |
| BFP | 0.0138914 | 0.00171911 | 6.40E-16 | rs957919 | T | C | 0.278023 | 65.29571782 |
| BFP | -0.0100803 | 0.00160438 | 3.30E-10 | rs4894808 | C | G | 0.40013 | 39.47593596 |
| BFP | -0.0120222 | 0.00168776 | 1.10E-12 | rs9865173 | A | T | 0.703199 | 50.73953904 |
| BFP | -0.0108516 | 0.0015618 | 3.70E-12 | rs7630228 | C | T | 0.434322 | 48.27660451 |
| BFP | -0.0198865 | 0.00308056 | 1.10E-10 | rs76345589 | G | C | 0.067492 | 41.6732494 |
| BFP | 0.0115052 | 0.00209731 | 4.10E-08 | rs28672845 | C | A | 0.839976 | 30.09283404 |
| BFP | -0.0137367 | 0.00229121 | 2.00E-09 | rs17016133 | C | T | 0.130053 | 35.94471422 |
| BFP | 0.0167794 | 0.00154077 | 1.30E-27 | rs9843653 | C | T | 0.51154 | 118.5979622 |
| BFP | -0.0121841 | 0.00165127 | 1.60E-13 | rs3911063 | C | T | 0.322 | 54.44408809 |
| BFP | -0.0135392 | 0.00153973 | 1.50E-18 | rs1454687 | G | C | 0.51535 | 77.32089351 |
| BFP | 0.00964481 | 0.00167852 | 9.10E-09 | rs1078455 | C | T | 0.309511 | 33.01674933 |
| BFP | -0.0136044 | 0.00161607 | 3.80E-17 | rs9814758 | G | T | 0.355786 | 70.8660863 |
| BFP | -0.0137926 | 0.00211992 | 7.70E-11 | rs2291127 | T | C | 0.156234 | 42.33049417 |
| BFP | 0.0117289 | 0.00158106 | 1.20E-13 | rs1568488 | C | G | 0.594863 | 55.03232539 |
| BFP | 0.0106966 | 0.00154338 | 4.20E-12 | rs529200 | G | A | 0.527782 | 48.03367055 |
| BFP | -0.0101996 | 0.00155143 | 4.90E-11 | rs6782581 | G | C | 0.439266 | 43.22170654 |
| BFP | 0.0110275 | 0.00189369 | 5.80E-09 | rs11129660 | T | C | 0.209783 | 33.91066928 |
| BFP | 0.0140939 | 0.00242625 | 6.30E-09 | rs1799923 | G | A | 0.886606 | 33.74358916 |
| BFP | -0.0161252 | 0.00262251 | 7.80E-10 | rs12639116 | T | C | 0.096558 | 37.80732181 |
| BFP | 0.0117998 | 0.00171613 | 6.20E-12 | rs2371767 | C | G | 0.277759 | 47.27689285 |
| BFP | 0.0123827 | 0.00158474 | 5.60E-15 | rs9289630 | C | G | 0.38912 | 61.05407594 |
| BFP | -0.0101838 | 0.00154798 | 4.70E-11 | rs4320040 | G | T | 0.560267 | 43.28017744 |
| BFP | -0.0108506 | 0.00190707 | 1.30E-08 | rs13064797 | A | G | 0.205187 | 32.37235087 |
| BFP | -0.0144347 | 0.00223876 | 1.10E-10 | rs75135487 | G | C | 0.136931 | 41.57195486 |
| BFP | -0.0152527 | 0.00204286 | 8.20E-14 | rs73213501 | C | A | 0.171839 | 55.74632559 |
| BFP | 0.0104262 | 0.00171657 | 1.20E-09 | rs28651380 | G | A | 0.281193 | 36.89173383 |
| BFP | -0.0104169 | 0.00155088 | 1.90E-11 | rs6843910 | A | T | 0.45449 | 45.11496235 |
| BFP | -0.021384 | 0.00368848 | 6.70E-09 | rs1086103 | A | C | 0.045775 | 33.61112319 |
| BFP | -0.0126754 | 0.00202574 | 3.90E-10 | rs4690324 | A | G | 0.823217 | 39.15217915 |
| BFP | -0.0102974 | 0.00185568 | 2.90E-08 | rs113503736 | G | T | 0.223372 | 30.79278659 |
| BFP | 0.0089709 | 0.00163023 | 3.70E-08 | rs7680610 | G | A | 0.652245 | 30.28128281 |
| BFP | 0.0121284 | 0.00154596 | 4.30E-15 | rs6840236 | C | T | 0.464881 | 61.54751429 |
| BFP | 0.0310059 | 0.00467505 | 3.30E-11 | rs1229984 | C | T | 0.972781 | 43.98617158 |
| BFP | 0.0201696 | 0.00155596 | 2.00E-38 | rs10938397 | G | A | 0.4343 | 168.0342124 |
| BFP | -0.0107277 | 0.00174038 | 7.10E-10 | rs3113509 | T | C | 0.731962 | 37.99487957 |
| BFP | -0.0123522 | 0.00154228 | 1.20E-15 | rs2276936 | C | A | 0.470368 | 64.14490648 |
| BFP | 0.0103136 | 0.00159872 | 1.10E-10 | rs34656389 | G | A | 0.367474 | 41.61747733 |
| BFP | 0.0323745 | 0.00292921 | 2.10E-28 | rs13107325 | T | C | 0.074871 | 122.1532773 |
| BFP | 0.0141172 | 0.00154567 | 6.60E-20 | rs2192527 | G | A | 0.465502 | 83.4187311 |
| BFP | -0.00936528 | 0.00161107 | 6.10E-09 | rs13132853 | G | A | 0.360225 | 33.79190778 |
| BFP | 0.0210464 | 0.00319585 | 4.50E-11 | rs112710809 | T | C | 0.063535 | 43.3693453 |
| BFP | 0.0113515 | 0.00161543 | 2.10E-12 | rs6847975 | A | G | 0.35617 | 49.37762752 |
| BFP | -0.010008 | 0.00161101 | 5.20E-10 | rs4398538 | C | T | 0.642513 | 38.59207418 |
| BFP | -0.0110497 | 0.0015726 | 2.10E-12 | rs1724557 | A | C | 0.586755 | 49.37014826 |
| BFP | -0.0133302 | 0.00160952 | 1.20E-16 | rs57800857 | C | A | 0.3649 | 68.59312286 |
| BFP | 0.0124473 | 0.00168847 | 1.70E-13 | rs17820010 | G | T | 0.295596 | 54.34550345 |
| BFP | -0.0102553 | 0.00178627 | 9.40E-09 | rs252749 | A | G | 0.245982 | 32.96116323 |
| BFP | 0.0113767 | 0.00154191 | 1.60E-13 | rs1503526 | C | T | 0.47999 | 54.43955443 |
| BFP | -0.014887 | 0.00201352 | 1.40E-13 | rs40071 | C | T | 0.179498 | 54.66413512 |
| BFP | 0.00988966 | 0.00156805 | 2.80E-10 | rs347551 | G | C | 0.472318 | 39.77799681 |
| BFP | 0.0133557 | 0.00173358 | 1.30E-14 | rs812949 | C | T | 0.729218 | 59.35339076 |
| BFP | -0.0100728 | 0.00154583 | 7.20E-11 | rs1991002 | G | T | 0.495176 | 42.45970368 |
| BFP | -0.0103073 | 0.00164616 | 3.80E-10 | rs10050620 | T | C | 0.325382 | 39.2053869 |
| BFP | -0.0126609 | 0.00160546 | 3.10E-15 | rs7707394 | A | G | 0.357305 | 62.19137775 |
| BFP | -0.0128176 | 0.00188108 | 9.50E-12 | rs7442885 | G | C | 0.214136 | 46.4300228 |
| BFP | 0.0245244 | 0.00226433 | 2.50E-27 | rs34483452 | A | C | 0.13631 | 117.3052207 |
| BFP | 0.011204 | 0.00164464 | 9.60E-12 | rs6875585 | C | A | 0.670422 | 46.40924496 |
| BFP | 0.0136415 | 0.0017496 | 6.30E-15 | rs13436840 | T | C | 0.265069 | 60.79203963 |
| BFP | -0.0308051 | 0.00388142 | 2.10E-15 | rs34580448 | C | T | 0.041248 | 62.98889174 |
| BFP | -0.0102893 | 0.00155919 | 4.10E-11 | rs396755 | G | C | 0.571071 | 43.54853788 |
| BFP | 0.0101719 | 0.00155683 | 6.40E-11 | rs7704382 | G | C | 0.433417 | 42.68957 |
| BFP | 0.0107194 | 0.0016852 | 2.00E-10 | rs12658319 | T | C | 0.299121 | 40.46113427 |
| BFP | -0.0111835 | 0.00176815 | 2.50E-10 | rs256904 | T | A | 0.746327 | 40.00527623 |
| BFP | -0.0135582 | 0.00241631 | 2.00E-08 | rs74288880 | T | C | 0.114819 | 31.48464273 |
| BFP | -0.0171461 | 0.00157693 | 1.50E-27 | rs2307111 | C | T | 0.39509 | 118.2240611 |
| BFP | 0.0128832 | 0.00218539 | 3.70E-09 | rs13174863 | G | A | 0.14814 | 34.7527835 |
| BFP | 0.0110461 | 0.00166011 | 2.90E-11 | rs2190788 | T | G | 0.319702 | 44.27353606 |
| BFP | 0.00897307 | 0.00164195 | 4.60E-08 | rs34338229 | A | G | 0.332008 | 29.86498187 |
| BFP | -0.0114888 | 0.00158746 | 4.60E-13 | rs1322842 | G | A | 0.609473 | 52.3773783 |
| BFP | 0.0126824 | 0.00214424 | 3.30E-09 | rs62413414 | T | C | 0.151703 | 34.982924 |
| BFP | -0.0117629 | 0.00202255 | 6.00E-09 | rs72995085 | C | T | 0.177033 | 33.82441363 |
| BFP | 0.00955622 | 0.00161789 | 3.50E-09 | rs4959613 | A | C | 0.594024 | 34.88785731 |
| BFP | -0.0232653 | 0.00172866 | 2.70E-41 | rs9358912 | T | G | 0.27332 | 181.133237 |
| BFP | -0.0106391 | 0.00156343 | 1.00E-11 | rs1624064 | C | T | 0.420353 | 46.30766678 |
| BFP | -0.0191217 | 0.002297 | 8.50E-17 | rs2178899 | T | A | 0.128827 | 69.29964505 |
| BFP | -0.00938942 | 0.00154401 | 1.20E-09 | rs998584 | A | C | 0.482871 | 36.98088622 |
| BFP | 0.0221404 | 0.00204337 | 2.30E-27 | rs72892910 | T | G | 0.172194 | 117.4023796 |
| BFP | -0.0108699 | 0.0019277 | 1.70E-08 | rs9321191 | C | T | 0.1994 | 31.79597909 |
| BFP | 0.00934269 | 0.00167115 | 2.30E-08 | rs4709745 | C | T | 0.307185 | 31.25453277 |
| BFP | -0.0138777 | 0.00190462 | 3.20E-13 | rs6927268 | G | T | 0.206332 | 53.09068257 |
| BFP | 0.010121 | 0.00167361 | 1.50E-09 | rs2182717 | A | G | 0.691021 | 36.57112543 |
| BFP | 0.0259158 | 0.00221742 | 1.50E-31 | rs2814993 | A | G | 0.13976 | 136.5945333 |
| BFP | -0.0109353 | 0.00188165 | 6.20E-09 | rs7773916 | T | C | 0.213136 | 33.77409452 |
| BFP | 0.0204619 | 0.0036589 | 2.20E-08 | rs35523808 | A | T | 0.049077 | 31.27453315 |
| BFP | -0.00933072 | 0.00162144 | 8.70E-09 | rs9372414 | T | C | 0.347629 | 33.11528896 |
| BFP | -0.0161687 | 0.00154706 | 1.40E-25 | rs1906252 | A | C | 0.484388 | 109.2284803 |
| BFP | 0.0136226 | 0.00165383 | 1.80E-16 | rs17681686 | C | G | 0.30483 | 67.84819181 |
| BFP | 0.0100294 | 0.00161777 | 5.70E-10 | rs2499468 | A | C | 0.650982 | 38.43406762 |
| BFP | -0.0120088 | 0.00210945 | 1.20E-08 | rs240999 | T | G | 0.840258 | 32.40863594 |
| BFP | 0.0122048 | 0.00154046 | 2.30E-15 | rs853961 | T | G | 0.505909 | 62.77120742 |
| BFP | 0.0198061 | 0.00346457 | 1.10E-08 | rs9389857 | T | C | 0.052433 | 32.68129476 |
| BFP | 0.00930584 | 0.00170461 | 4.80E-08 | rs7762794 | G | A | 0.285523 | 29.80307658 |
| BFP | -0.0140163 | 0.00215528 | 7.90E-11 | rs55810445 | T | C | 0.151196 | 42.29211175 |
| BFP | 0.0113141 | 0.00203645 | 2.80E-08 | rs7796825 | A | G | 0.825769 | 30.86686502 |
| BFP | -0.0139877 | 0.00189659 | 1.60E-13 | rs10259620 | G | A | 0.787159 | 54.3933373 |
| BFP | -0.010769 | 0.0015641 | 5.80E-12 | rs17172722 | T | C | 0.418942 | 47.40473486 |
| BFP | -0.0105935 | 0.00177956 | 2.60E-09 | rs6948959 | A | G | 0.744356 | 35.43673257 |
| BFP | -0.00954008 | 0.00162185 | 4.00E-09 | rs4496901 | T | G | 0.65522 | 34.60052296 |
| BFP | 0.0143436 | 0.00224286 | 1.60E-10 | rs4722398 | T | C | 0.13612 | 40.89893506 |
| BFP | 0.00928324 | 0.00157441 | 3.70E-09 | rs6973656 | G | A | 0.397021 | 34.76670061 |
| BFP | -0.0138378 | 0.00213519 | 9.10E-11 | rs10271582 | G | A | 0.154048 | 42.0011368 |
| BFP | -0.0118159 | 0.00164828 | 7.60E-13 | rs6977416 | A | G | 0.334244 | 51.38917382 |
| BFP | -0.00992339 | 0.00174424 | 1.30E-08 | rs798549 | A | C | 0.730794 | 32.36738669 |
| BFP | -0.012096 | 0.00158845 | 2.60E-14 | rs215669 | A | G | 0.611659 | 57.98777676 |
| BFP | 0.00852919 | 0.00155109 | 3.80E-08 | rs2192649 | G | T | 0.500777 | 30.23720068 |
| BFP | -0.0146575 | 0.00217921 | 1.70E-11 | rs17704028 | T | C | 0.147192 | 45.23990033 |
| BFP | -0.0127186 | 0.00155559 | 2.90E-16 | rs7789056 | A | G | 0.563037 | 66.84798682 |
| BFP | -0.0109846 | 0.00175319 | 3.70E-10 | rs12538435 | G | A | 0.261821 | 39.25640487 |
| BFP | -0.0131217 | 0.00156604 | 5.30E-17 | rs58862095 | T | C | 0.419262 | 70.20604487 |
| BFP | -0.0143133 | 0.00154037 | 1.50E-20 | rs972283 | G | A | 0.511944 | 86.34345929 |
| BFP | -0.0106141 | 0.00155316 | 8.30E-12 | rs62443626 | A | G | 0.463946 | 46.70183612 |
| BFP | 0.0111105 | 0.00157055 | 1.50E-12 | rs4718964 | T | G | 0.413126 | 50.04534334 |
| BFP | 0.0102996 | 0.00156914 | 5.20E-11 | rs12375196 | A | C | 0.424277 | 43.08413026 |
| BFP | 0.00941089 | 0.00168003 | 2.10E-08 | rs12670456 | G | A | 0.301754 | 31.37814897 |
| BFP | 0.00917484 | 0.00167492 | 4.30E-08 | rs10245306 | C | G | 0.685981 | 30.00605229 |
| BFP | 0.0112967 | 0.00154993 | 3.10E-13 | rs11786089 | G | A | 0.459661 | 53.12256326 |
| BFP | 0.0152146 | 0.00155645 | 1.40E-22 | rs10100245 | A | G | 0.564497 | 95.55440893 |
| BFP | 0.00986943 | 0.00170532 | 7.10E-09 | rs1559900 | T | C | 0.286141 | 33.49441301 |
| BFP | 0.0124143 | 0.00167398 | 1.20E-13 | rs2954033 | G | A | 0.695224 | 54.9976259 |
| BFP | 0.0172978 | 0.00261256 | 3.60E-11 | rs117176448 | G | C | 0.096175 | 43.83784496 |
| BFP | 0.0173998 | 0.00171871 | 4.30E-24 | rs4876611 | G | A | 0.720188 | 102.4904276 |
| BFP | -0.01632 | 0.00166697 | 1.20E-22 | rs1808629 | A | G | 0.685441 | 95.84837202 |
| BFP | 0.0103333 | 0.00170753 | 1.40E-09 | rs1813039 | A | G | 0.709746 | 36.62194373 |
| BFP | 0.00954133 | 0.00161413 | 3.40E-09 | rs13249935 | C | T | 0.366657 | 34.94144127 |
| BFP | 0.0115499 | 0.00194851 | 3.10E-09 | rs72703757 | C | G | 0.198824 | 35.1359071 |
| BFP | -0.0101175 | 0.00165029 | 8.70E-10 | rs28714450 | T | C | 0.675249 | 37.58598101 |
| BFP | -0.0113003 | 0.00155717 | 4.00E-13 | rs1421334 | C | A | 0.54884 | 52.66327827 |
| BFP | 0.0088055 | 0.00154188 | 1.10E-08 | rs7463186 | G | A | 0.516044 | 32.6142122 |
| BFP | 0.0103657 | 0.00166877 | 5.20E-10 | rs7843109 | T | C | 0.688667 | 38.58373833 |
| BFP | 0.00997781 | 0.00155638 | 1.40E-10 | rs4466418 | A | G | 0.562278 | 41.09974968 |
| BFP | 0.0106691 | 0.00161125 | 3.60E-11 | rs11782074 | T | G | 0.383624 | 43.84597286 |
| BFP | -0.0219296 | 0.00320576 | 7.90E-12 | rs10116857 | A | C | 0.061826 | 46.7949954 |
| BFP | 0.011578 | 0.0016271 | 1.10E-12 | rs7027304 | T | C | 0.652657 | 50.63357597 |
| BFP | -0.0123455 | 0.00207928 | 2.90E-09 | rs55924785 | T | C | 0.164678 | 35.25262126 |
| BFP | -0.0140265 | 0.00160866 | 2.80E-18 | rs10756798 | T | C | 0.642437 | 76.02739429 |
| BFP | 0.0154638 | 0.00164622 | 5.80E-21 | rs17770336 | T | C | 0.322448 | 88.23821235 |
| BFP | 0.0124051 | 0.00185992 | 2.60E-11 | rs41307479 | G | C | 0.220577 | 44.48483767 |
| BFP | -0.0110309 | 0.00181676 | 1.30E-09 | rs12376870 | A | G | 0.237663 | 36.86606431 |
| BFP | -0.00914252 | 0.00165304 | 3.20E-08 | rs7046679 | G | C | 0.678548 | 30.58898826 |
| BFP | -0.0111522 | 0.00196791 | 1.50E-08 | rs10867315 | A | G | 0.190594 | 32.11520018 |
| BFP | 0.0102177 | 0.00154181 | 3.40E-11 | rs3923501 | T | C | 0.475818 | 43.91820467 |
| BFP | -0.0143754 | 0.00240082 | 2.10E-09 | rs16916303 | G | A | 0.11977 | 35.8526016 |
| BFP | 0.0116159 | 0.00154667 | 5.90E-14 | rs7357754 | G | A | 0.500129 | 56.40406515 |
| BFP | -0.00911255 | 0.00158643 | 9.20E-09 | rs10959841 | C | T | 0.387368 | 32.99423176 |
| BFP | -0.0200044 | 0.0015583 | 1.00E-37 | rs13292699 | C | A | 0.433725 | 164.7968547 |
| BFP | -0.0101722 | 0.00154703 | 4.90E-11 | rs4837119 | A | T | 0.517271 | 43.23468605 |
| BFP | 0.016885 | 0.00175383 | 6.10E-22 | rs10999460 | T | C | 0.265521 | 92.68877464 |
| BFP | -0.0110832 | 0.00156255 | 1.30E-12 | rs2172131 | C | T | 0.578543 | 50.31094724 |
| BFP | 0.0102433 | 0.00157002 | 6.80E-11 | rs2002023 | T | C | 0.409059 | 42.56664436 |
| BFP | 0.0092676 | 0.00164764 | 1.90E-08 | rs482787 | C | T | 0.325766 | 31.63806457 |
| BFP | -0.0201214 | 0.00257596 | 5.70E-15 | rs41310284 | A | C | 0.101004 | 61.01521689 |
| BFP | 0.0128932 | 0.00215901 | 2.30E-09 | rs16934748 | C | T | 0.150341 | 35.66252846 |
| BFP | -0.00953851 | 0.00156462 | 1.10E-09 | rs6480350 | C | T | 0.575106 | 37.16578617 |
| BFP | 0.0129238 | 0.00179232 | 5.60E-13 | rs10510025 | T | C | 0.24708 | 51.99353612 |
| BFP | 0.00937385 | 0.00156054 | 1.90E-09 | rs11245344 | T | C | 0.571137 | 36.08163337 |
| BFP | 0.0113458 | 0.00171702 | 3.90E-11 | rs67609008 | C | T | 0.283593 | 43.66359998 |
| BFP | 0.0100326 | 0.00163856 | 9.20E-10 | rs7893571 | T | G | 0.665915 | 37.48886542 |
| BFP | 0.0103873 | 0.00154427 | 1.70E-11 | rs10788497 | C | G | 0.499616 | 45.24377984 |
| BFP | -0.0159065 | 0.00155477 | 1.40E-24 | rs2274224 | C | G | 0.435459 | 104.6687302 |
| BFP | 0.0166039 | 0.00163819 | 3.80E-24 | rs11012732 | G | A | 0.331719 | 102.7286728 |
| BFP | 0.0222563 | 0.0015648 | 6.60E-46 | rs7124681 | A | C | 0.408419 | 202.2964485 |
| BFP | -0.0115343 | 0.0015826 | 3.10E-13 | rs719802 | C | T | 0.6144 | 53.11781024 |
| BFP | -0.0142941 | 0.00208312 | 6.80E-12 | rs61910767 | T | C | 0.164197 | 47.08526889 |
| BFP | 0.0132564 | 0.001761 | 5.20E-14 | rs11030016 | T | C | 0.739777 | 56.66729578 |
| BFP | -0.00954107 | 0.00161396 | 3.40E-09 | rs685149 | G | A | 0.645411 | 34.94689782 |
| BFP | -0.0144775 | 0.00166392 | 3.30E-18 | rs479018 | A | G | 0.332499 | 75.704599 |
| BFP | -0.0105396 | 0.00189331 | 2.60E-08 | rs7942368 | T | C | 0.217538 | 30.98880293 |
| BFP | 0.0129238 | 0.00209124 | 6.40E-10 | rs1605898 | A | T | 0.837607 | 38.19202647 |
| BFP | 0.00937803 | 0.00155792 | 1.70E-09 | rs2508782 | G | A | 0.569716 | 36.23538917 |
| BFP | 0.0118153 | 0.00156853 | 5.00E-14 | rs11222371 | T | C | 0.409234 | 56.74189652 |
| BFP | -0.0191979 | 0.00335242 | 1.00E-08 | rs1881505 | C | T | 0.943033 | 32.79371397 |
| BFP | -0.0107504 | 0.00193009 | 2.50E-08 | rs11022718 | T | C | 0.202968 | 31.02373816 |
| BFP | -0.00913519 | 0.00162015 | 1.70E-08 | rs1782508 | G | C | 0.655489 | 31.79250284 |
| BFP | 0.0170384 | 0.00167907 | 3.40E-24 | rs59227842 | G | A | 0.311468 | 102.9722056 |
| BFP | -0.00989059 | 0.00154908 | 1.70E-10 | rs657685 | C | G | 0.456088 | 40.76586901 |
| BFP | -0.0146358 | 0.00172806 | 2.50E-17 | rs885114 | A | G | 0.275581 | 71.73238937 |
| BFP | 0.0116378 | 0.00154184 | 4.40E-14 | rs2957678 | C | T | 0.498199 | 56.97222254 |
| BFP | -0.0185028 | 0.00165098 | 3.80E-29 | rs11030108 | G | A | 0.679962 | 125.6004758 |
| BFP | 0.0375397 | 0.00633223 | 3.10E-09 | rs55707359 | G | T | 0.015449 | 35.14537918 |
| BFP | 0.0115223 | 0.00187372 | 7.80E-10 | rs10896012 | C | T | 0.217394 | 37.81542382 |
| BFP | 0.0118139 | 0.00177153 | 2.60E-11 | rs61903695 | G | A | 0.254879 | 44.47229667 |
| BFP | 0.0206723 | 0.00333136 | 5.50E-10 | rs12419272 | G | C | 0.056932 | 38.5065371 |
| BFP | 0.0111592 | 0.00156858 | 1.10E-12 | rs7925725 | C | A | 0.411245 | 50.61191569 |
| BFP | 0.0128411 | 0.00223802 | 9.60E-09 | rs10505836 | C | A | 0.860003 | 32.92126613 |
| BFP | 0.0104428 | 0.00160707 | 8.10E-11 | rs4759318 | T | C | 0.362201 | 42.22448225 |
| BFP | -0.0218753 | 0.00336997 | 8.50E-11 | rs2731238 | T | G | 0.067529 | 42.13625813 |
| BFP | -0.0101643 | 0.00160647 | 2.50E-10 | rs11105842 | A | G | 0.367542 | 40.03222326 |
| BFP | 0.0167619 | 0.00175434 | 1.20E-21 | rs7972728 | A | C | 0.73765 | 91.28910713 |
| BFP | 0.00983786 | 0.00163453 | 1.80E-09 | rs2108635 | G | A | 0.338522 | 36.22559137 |
| BFP | 0.0197321 | 0.00158551 | 1.50E-35 | rs7132908 | A | G | 0.38447 | 154.8847448 |
| BFP | 0.0115537 | 0.00160056 | 5.30E-13 | rs2111281 | C | A | 0.366942 | 52.10726216 |
| BFP | -0.0216364 | 0.00347856 | 5.00E-10 | rs75412871 | T | C | 0.051916 | 38.68753027 |
| BFP | 0.0185779 | 0.00165616 | 3.30E-29 | rs7133378 | A | G | 0.319439 | 125.831293 |
| BFP | -0.0186517 | 0.00175335 | 2.00E-26 | rs3764002 | T | C | 0.261517 | 113.1617377 |
| BFP | -0.0204973 | 0.002693 | 2.70E-14 | rs74576293 | C | T | 0.089728 | 57.93227829 |
| BFP | -0.0113662 | 0.00186311 | 1.10E-09 | rs4762951 | G | A | 0.780444 | 37.21804816 |
| BFP | 0.00973719 | 0.00154554 | 3.00E-10 | rs1350429 | G | A | 0.480306 | 39.69234795 |
| BFP | -0.0153041 | 0.00266658 | 9.50E-09 | rs11062595 | G | C | 0.09222 | 32.93869241 |
| BFP | 0.00918558 | 0.00164856 | 2.50E-08 | rs7960609 | G | A | 0.326919 | 31.04585692 |
| BFP | 0.0115294 | 0.00188095 | 8.80E-10 | rs7975187 | G | A | 0.213862 | 37.57153263 |
| BFP | 0.0312346 | 0.00554241 | 1.70E-08 | rs61754230 | T | C | 0.019711 | 31.75956885 |
| BFP | 0.0136927 | 0.00154796 | 9.10E-19 | rs704061 | C | T | 0.455038 | 78.2453825 |
| BFP | -0.00981406 | 0.00177103 | 3.00E-08 | rs7966251 | A | G | 0.255023 | 30.70757928 |
| BFP | -0.0132887 | 0.00173423 | 1.80E-14 | rs78296744 | A | G | 0.272344 | 58.71534334 |
| BFP | -0.0108221 | 0.00168444 | 1.30E-10 | rs11619722 | C | T | 0.300933 | 41.27736763 |
| BFP | 0.0193443 | 0.00231898 | 7.30E-17 | rs9568867 | A | G | 0.129188 | 69.58442317 |
| BFP | -0.013993 | 0.00170162 | 2.00E-16 | rs6491427 | G | A | 0.289013 | 67.62332258 |
| BFP | -0.0139023 | 0.00241521 | 8.60E-09 | rs76115890 | C | T | 0.117876 | 33.13321019 |
| BFP | -0.0118589 | 0.00155668 | 2.60E-14 | rs56399737 | T | C | 0.44917 | 58.03501786 |
| BFP | 0.0107397 | 0.00177343 | 1.40E-09 | rs7321331 | A | G | 0.742296 | 36.67382416 |
| BFP | -0.0123469 | 0.00200425 | 7.30E-10 | rs17055384 | T | C | 0.182952 | 37.95002592 |
| BFP | 0.0127422 | 0.00173502 | 2.10E-13 | rs61969510 | C | T | 0.279026 | 53.93614021 |
| BFP | 0.0146332 | 0.00219293 | 2.50E-11 | rs11619393 | C | T | 0.144968 | 44.52757861 |
| BFP | -0.0138119 | 0.00193112 | 8.50E-13 | rs7987928 | A | G | 0.799871 | 51.15502806 |
| BFP | -0.0117153 | 0.00179629 | 6.90E-11 | rs6561937 | A | T | 0.753684 | 42.5357333 |
| BFP | 0.0119959 | 0.00160493 | 7.80E-14 | rs1441264 | A | G | 0.59371 | 55.86675975 |
| BFP | -0.0120426 | 0.00163641 | 1.90E-13 | rs2243928 | G | C | 0.648983 | 54.15720878 |
| BFP | 0.00930927 | 0.00156135 | 2.50E-09 | rs2481899 | G | A | 0.551171 | 35.54927252 |
| BFP | 0.0120832 | 0.00161524 | 7.40E-14 | rs12890931 | G | T | 0.362416 | 55.96155915 |
| BFP | 0.0133993 | 0.00160775 | 7.80E-17 | rs6575340 | A | G | 0.636023 | 69.45878545 |
| BFP | 0.0189406 | 0.00283307 | 2.30E-11 | rs61986205 | G | A | 0.082024 | 44.69643184 |
| BFP | -0.052548 | 0.00748992 | 2.30E-12 | rs72681698 | C | T | 0.010721 | 49.22186041 |
| BFP | 0.0129295 | 0.00173907 | 1.00E-13 | rs217672 | C | A | 0.27173 | 55.27507421 |
| BFP | 0.00996462 | 0.00154621 | 1.20E-10 | rs12432026 | G | T | 0.540113 | 41.53216089 |
| BFP | -0.0153167 | 0.00203008 | 4.50E-14 | rs72697297 | C | T | 0.179917 | 56.92513995 |
| BFP | 0.018731 | 0.00185697 | 6.30E-24 | rs10146997 | G | A | 0.221871 | 101.7447836 |
| BFP | 0.0100925 | 0.00158231 | 1.80E-10 | rs10144067 | T | C | 0.59128 | 40.68313114 |
| BFP | -0.0158356 | 0.00179488 | 1.10E-18 | rs9788550 | C | G | 0.24751 | 77.83917237 |
| BFP | -0.0142041 | 0.00207301 | 7.30E-12 | rs61975147 | C | T | 0.167185 | 46.94881642 |
| BFP | -0.0115932 | 0.00163624 | 1.40E-12 | rs3803286 | G | A | 0.666725 | 50.20103205 |
| BFP | 0.0126085 | 0.00155155 | 4.40E-16 | rs17522122 | T | G | 0.471197 | 66.03821019 |
| BFP | 0.0117936 | 0.00178131 | 3.60E-11 | rs2165991 | G | A | 0.253707 | 43.83426974 |
| BFP | 0.0133595 | 0.00155278 | 7.70E-18 | rs2415142 | G | T | 0.548783 | 74.02195503 |
| BFP | -0.0152755 | 0.00174905 | 2.50E-18 | rs3817428 | G | C | 0.265007 | 76.2757382 |
| BFP | -0.012668 | 0.00193068 | 5.30E-11 | rs72767957 | G | A | 0.200131 | 43.05221634 |
| BFP | 0.0114053 | 0.0017089 | 2.50E-11 | rs12441543 | A | G | 0.287112 | 44.54306803 |
| BFP | 0.0209909 | 0.00297573 | 1.70E-12 | rs113941571 | T | C | 0.078721 | 49.75939285 |
| BFP | -0.021134 | 0.00191016 | 1.90E-28 | rs28742003 | T | C | 0.205276 | 122.4119774 |
| BFP | -0.0127741 | 0.00231146 | 3.30E-08 | rs149380583 | C | G | 0.128655 | 30.54132388 |
| BFP | 0.0226029 | 0.00170655 | 4.80E-40 | rs6602997 | T | C | 0.711856 | 175.4245184 |
| BFP | 0.00998636 | 0.00154808 | 1.10E-10 | rs4776337 | A | G | 0.468075 | 41.61286678 |
| BFP | -0.0103933 | 0.00174766 | 2.70E-09 | rs11855853 | T | C | 0.269414 | 35.36657747 |
| BFP | -0.0163172 | 0.0024497 | 2.70E-11 | rs72755233 | A | G | 0.11145 | 44.36755221 |
| BFP | 0.0106498 | 0.00175622 | 1.30E-09 | rs11852419 | T | A | 0.261669 | 36.77266172 |
| BFP | -0.0156141 | 0.0027727 | 1.80E-08 | rs72803260 | G | T | 0.084979 | 31.71232971 |
| BFP | 0.041033 | 0.00157131 | 2.50E-150 | rs56094641 | G | A | 0.404646 | 681.9347026 |
| BFP | -0.00977478 | 0.00160607 | 1.20E-09 | rs4500770 | T | A | 0.362615 | 37.04119961 |
| BFP | 0.014719 | 0.00158851 | 1.90E-20 | rs879620 | T | C | 0.613285 | 85.85719669 |
| BFP | 0.0110602 | 0.00179199 | 6.70E-10 | rs6500594 | G | T | 0.246636 | 38.09384383 |
| BFP | 0.0108857 | 0.00160421 | 1.20E-11 | rs2660241 | C | T | 0.36485 | 46.04582773 |
| BFP | -0.0111483 | 0.00189749 | 4.20E-09 | rs2966859 | G | A | 0.789007 | 34.51900768 |
| BFP | -0.00918915 | 0.00161862 | 1.40E-08 | rs12926311 | C | G | 0.353546 | 32.23004241 |
| BFP | 0.0232064 | 0.00157608 | 4.50E-49 | rs7498665 | G | A | 0.399562 | 216.799877 |
| BFP | -0.0147342 | 0.00159151 | 2.10E-20 | rs881929 | T | G | 0.375396 | 85.71056889 |
| BFP | -0.0158787 | 0.00158501 | 1.30E-23 | rs11866219 | C | A | 0.583787 | 100.3612066 |
| BFP | 0.00867343 | 0.00158474 | 4.40E-08 | rs11343 | G | T | 0.561331 | 29.95475113 |
| BFP | 0.0104802 | 0.00155885 | 1.80E-11 | rs12103006 | G | A | 0.569085 | 45.19917279 |
| BFP | -0.00952112 | 0.00165613 | 9.00E-09 | rs56369689 | G | A | 0.348594 | 33.05119977 |
| BFP | 0.0095458 | 0.00155694 | 8.70E-10 | rs811054 | T | C | 0.537276 | 37.59074293 |
| BFP | 0.0101676 | 0.00165236 | 7.60E-10 | rs7206608 | G | C | 0.321575 | 37.86409305 |
| BFP | -0.0103504 | 0.00156479 | 3.70E-11 | rs3743861 | C | G | 0.415256 | 43.75242646 |
| BFP | -0.0208407 | 0.00214396 | 2.50E-22 | rs4790841 | T | C | 0.154504 | 94.49117449 |
| BFP | 0.0113474 | 0.00175799 | 1.10E-10 | rs78744936 | A | G | 0.265987 | 41.66390072 |
| BFP | -0.0109061 | 0.00161091 | 1.30E-11 | rs7216121 | G | A | 0.626339 | 45.83491138 |
| BFP | 0.0153102 | 0.00181772 | 3.70E-17 | rs2855818 | A | G | 0.241541 | 70.94270709 |
| BFP | -0.0130416 | 0.00164632 | 2.30E-15 | rs11079849 | T | C | 0.328562 | 62.75281946 |
| BFP | 0.0100634 | 0.00163774 | 8.00E-10 | rs9892466 | A | T | 0.333275 | 37.75718067 |
| BFP | 0.00883995 | 0.00158047 | 2.20E-08 | rs319775 | C | T | 0.60881 | 31.28434769 |
| BFP | 0.0211077 | 0.00194282 | 1.70E-27 | rs7218014 | C | T | 0.197302 | 118.036601 |
| BFP | 0.0119487 | 0.00190099 | 3.30E-10 | rs2034946 | G | T | 0.208834 | 39.50769115 |
| BFP | 0.00915425 | 0.00154807 | 3.40E-09 | rs3826408 | T | C | 0.456883 | 34.96748106 |
| BFP | -0.0113255 | 0.00185836 | 1.10E-09 | rs1863115 | A | C | 0.741492 | 37.14112641 |
| BFP | 0.0125471 | 0.00154189 | 4.00E-16 | rs1038088 | G | T | 0.519335 | 66.21859091 |
| BFP | 0.0110009 | 0.00164659 | 2.40E-11 | rs2008018 | A | G | 0.322077 | 44.63602238 |
| BFP | -0.0126783 | 0.00166019 | 2.20E-14 | rs11150745 | G | A | 0.317696 | 58.31851556 |
| BFP | -0.00942576 | 0.00168416 | 2.20E-08 | rs10513935 | A | G | 0.301501 | 31.3231953 |
| BFP | 0.010994 | 0.00163476 | 1.80E-11 | rs11664106 | T | A | 0.373948 | 45.2275914 |
| BFP | -0.00962166 | 0.0016001 | 1.80E-09 | rs1945160 | A | G | 0.375871 | 36.15811336 |
| BFP | -0.0137829 | 0.00163035 | 2.80E-17 | rs59499656 | T | A | 0.343189 | 71.46929775 |
| BFP | 0.00965173 | 0.00163558 | 3.60E-09 | rs11664848 | G | C | 0.659616 | 34.82304516 |
| BFP | 0.0254579 | 0.00182642 | 3.70E-44 | rs6567160 | C | T | 0.232676 | 194.2870455 |
| BFP | 0.0127569 | 0.00220651 | 7.40E-09 | rs9955276 | T | C | 0.144431 | 33.42554539 |
| BFP | 0.0107485 | 0.00169686 | 2.40E-10 | rs8096564 | T | G | 0.298122 | 40.12395154 |
| BFP | 0.00961178 | 0.00155008 | 5.60E-10 | rs1787013 | C | T | 0.450716 | 38.45027216 |
| BFP | -0.0163989 | 0.00155688 | 6.10E-26 | rs1893659 | A | C | 0.460098 | 110.9479177 |
| BFP | -0.0252015 | 0.00279375 | 1.90E-19 | rs57636386 | C | T | 0.083836 | 81.37250783 |
| BFP | -0.0171142 | 0.00198547 | 6.70E-18 | rs72976986 | A | G | 0.190192 | 74.29961223 |
| BFP | -0.0249637 | 0.00423712 | 3.80E-09 | rs62621197 | T | C | 0.037191 | 34.71173982 |
| BFP | -0.0215029 | 0.00213952 | 9.20E-24 | rs429358 | C | T | 0.154044 | 101.0093018 |
| BFP | 0.00878996 | 0.00154865 | 1.40E-08 | rs1469084 | G | A | 0.545742 | 32.21567563 |
| BFP | -0.0126806 | 0.00174938 | 4.20E-13 | rs12459965 | T | C | 0.267624 | 52.54256768 |
| BFP | -0.0143589 | 0.00158003 | 1.00E-19 | rs8112818 | G | A | 0.400376 | 82.58699723 |
| BFP | -0.0232502 | 0.00194747 | 7.40E-33 | rs10423928 | A | T | 0.194436 | 142.531821 |
| BFP | -0.0177091 | 0.00160075 | 1.90E-28 | rs11666808 | C | T | 0.625293 | 122.390007 |
| BFP | -0.0163804 | 0.00156772 | 1.50E-25 | rs33836 | T | C | 0.464327 | 109.1721864 |
| BFP | 0.0118765 | 0.00182093 | 6.90E-11 | rs9304665 | A | T | 0.763642 | 42.53931028 |
| BFP | 0.0123462 | 0.00165332 | 8.20E-14 | rs12462975 | A | G | 0.32959 | 55.76385345 |
| BFP | -0.0147849 | 0.00232906 | 2.20E-10 | rs6103254 | C | T | 0.126684 | 40.29725183 |
| BFP | -0.0128916 | 0.00188796 | 8.60E-12 | rs56218501 | T | C | 0.211814 | 46.62598999 |
| BFP | -0.0130773 | 0.0016559 | 2.80E-15 | rs6021948 | A | T | 0.32177 | 62.36888231 |
| BFP | -0.0105166 | 0.00185641 | 1.50E-08 | rs6064113 | C | T | 0.765465 | 32.09245295 |
| BFP | 0.0194862 | 0.00316613 | 7.50E-10 | rs843901 | G | T | 0.936414 | 37.87885352 |
| BFP | 0.0140368 | 0.00231882 | 1.40E-09 | rs16996657 | C | T | 0.127757 | 36.64393878 |
| BFP | 0.0130676 | 0.00155587 | 4.50E-17 | rs7020 | A | G | 0.437471 | 70.54155853 |
| BFP | -0.0164403 | 0.00215064 | 2.10E-14 | rs112852122 | A | G | 0.157829 | 58.43647874 |
| BFP | 0.0120757 | 0.0016495 | 2.50E-13 | rs1056441 | C | T | 0.675301 | 53.59447125 |
| BFP | -0.0127956 | 0.00208683 | 8.70E-10 | rs62218301 | G | A | 0.166049 | 37.59647844 |
| BFP | 0.0123902 | 0.00155475 | 1.60E-15 | rs394608 | C | T | 0.537747 | 63.50903334 |
| BFP | -0.0180452 | 0.00314532 | 9.60E-09 | rs17193211 | T | C | 0.066803 | 32.91498994 |
| BFP | 0.00875321 | 0.00155205 | 1.70E-08 | rs1475860 | C | G | 0.505368 | 31.80704178 |
| BFP | 0.0132675 | 0.00213178 | 4.90E-10 | rs74618095 | C | T | 0.157976 | 38.73409611 |
| BFP | -0.00959714 | 0.00159107 | 1.60E-09 | rs12628603 | A | G | 0.617054 | 36.38355123 |
| BFP | -0.0169383 | 0.00156868 | 3.50E-27 | rs4820323 | G | C | 0.580958 | 116.5925825 |
| BFP | 0.0121586 | 0.00204591 | 2.80E-09 | rs11538 | G | A | 0.171838 | 35.31783652 |
| BFP | 0.0104441 | 0.00155077 | 1.60E-11 | rs10854853 | T | G | 0.456784 | 45.35730698 |
| BMI | -0.0168 | 0.003 | 2.18E-08 | rs977747 | G | T | 0.5333 | 31.36 |
| BMI | 0.0201 | 0.0031 | 4.57E-11 | rs17381664 | C | T | 0.425 | 42.04058273 |
| BMI | 0.0659 | 0.0087 | 5.06E-14 | rs7550711 | T | C | 0.0339 | 57.37627163 |
| BMI | 0.0181 | 0.0029 | 5.45E-10 | rs2820292 | C | A | 0.5083 | 38.9548157 |
| BMI | 0.0331 | 0.003 | 1.88E-28 | rs7531118 | C | T | 0.6083 | 121.7344444 |
| BMI | 0.0497 | 0.0037 | 2.29E-40 | rs543874 | G | A | 0.2667 | 180.4302411 |
| BMI | -0.0227 | 0.0031 | 2.12E-13 | rs657452 | G | A | 0.5833 | 53.6201873 |
| BMI | 0.0221 | 0.003 | 1.43E-13 | rs11165643 | T | C | 0.575 | 54.26777778 |
| BMI | 0.0209 | 0.0038 | 4.98E-08 | rs1460676 | C | T | 0.2167 | 30.25 |
| BMI | 0.0175 | 0.003 | 4.77E-09 | rs1528435 | T | C | 0.5833 | 34.02777778 |
| BMI | -0.0228 | 0.0033 | 4.36E-12 | rs1016287 | C | T | 0.675 | 47.73553719 |
| BMI | 0.0279 | 0.0049 | 1.24E-08 | rs2890652 | C | T | 0.125 | 32.42024157 |
| BMI | 0.0604 | 0.0039 | 5.44E-54 | rs13021737 | G | A | 0.875 | 239.8527285 |
| BMI | 0.0164 | 0.0029 | 1.97E-08 | rs6713510 | A | G | 0.4833 | 31.98097503 |
| BMI | 0.0309 | 0.0029 | 8.07E-26 | rs10182181 | G | A | 0.5 | 113.5326992 |
| BMI | 0.0207 | 0.0036 | 8.92E-09 | rs12986742 | C | T | 0.5 | 33.0625 |
| BMI | 0.0211 | 0.0038 | 3.41E-08 | rs17203016 | G | A | 0.2 | 30.83171745 |
| BMI | -0.0214 | 0.0033 | 4.73E-11 | rs7599312 | A | G | 0.2917 | 42.05325987 |
| BMI | 0.0183 | 0.0033 | 1.93E-08 | rs3849570 | A | C | 0.3667 | 30.75206612 |
| BMI | 0.0183 | 0.003 | 8.02E-10 | rs6804842 | G | A | 0.575 | 37.21 |
| BMI | -0.0195 | 0.003 | 1.35E-10 | rs2365389 | T | C | 0.3417 | 42.25 |
| BMI | 0.029 | 0.0038 | 1.42E-14 | rs13078960 | G | T | 0.1833 | 58.24099723 |
| BMI | 0.0478 | 0.0075 | 1.85E-10 | rs16851483 | T | G | 0.0917 | 40.61937778 |
| BMI | 0.0448 | 0.0044 | 1.39E-24 | rs1516725 | C | T | 0.9083 | 103.6694215 |
| BMI | 0.0304 | 0.0052 | 5.03E-09 | rs17001654 | G | C | 0.1583 | 34.17751479 |
| BMI | 0.0398 | 0.003 | 8.01E-41 | rs13130484 | T | C | 0.4333 | 176.0044444 |
| BMI | 0.0472 | 0.0066 | 1.06E-12 | rs13107325 | T | C | 0.1167 | 51.14416896 |
| BMI | -0.0365 | 0.0063 | 6.25E-09 | rs11727676 | C | T | 0.075 | 33.56638952 |
| BMI | -0.0254 | 0.003 | 1.96E-17 | rs2112347 | G | T | 0.375 | 71.68444444 |
| BMI | -0.0168 | 0.0029 | 8.85E-09 | rs7715256 | T | G | 0.55 | 33.56004756 |
| BMI | 0.0209 | 0.0033 | 2.54E-10 | rs6457796 | C | T | 0.2583 | 40.11111111 |
| BMI | 0.0444 | 0.0038 | 4.52E-31 | rs943005 | T | C | 0.1 | 136.5207756 |
| BMI | 0.0183 | 0.0032 | 1.45E-08 | rs2033529 | G | A | 0.2583 | 32.70410156 |
| BMI | 0.0236 | 0.0043 | 4.28E-08 | rs13201877 | G | A | 0.0833 | 30.12222823 |
| BMI | -0.0285 | 0.0047 | 1.09E-09 | rs13191362 | G | A | 0.2 | 36.77003169 |
| BMI | 0.0196 | 0.0034 | 7.20E-09 | rs9374842 | T | C | 0.7417 | 33.23183391 |
| BMI | 0.0175 | 0.0032 | 4.95E-08 | rs3800229 | T | G | 0.6917 | 29.90722656 |
| BMI | 0.02 | 0.0031 | 1.98E-10 | rs1167827 | G | A | 0.5417 | 41.62330905 |
| BMI | -0.0203 | 0.003 | 9.46E-12 | rs2060604 | C | T | 0.4417 | 45.78777778 |
| BMI | 0.0241 | 0.0032 | 2.22E-14 | rs2183825 | C | T | 0.2917 | 56.71972656 |
| BMI | -0.017 | 0.0029 | 6.36E-09 | rs4740619 | C | T | 0.4667 | 34.36385256 |
| BMI | -0.0182 | 0.0029 | 4.32E-10 | rs1928295 | C | T | 0.425 | 39.38644471 |
| BMI | -0.0169 | 0.003 | 1.70E-08 | rs6477694 | T | C | 0.6417 | 31.73444444 |
| BMI | -0.0188 | 0.003 | 2.45E-10 | rs10733682 | G | A | 0.575 | 39.27111111 |
| BMI | -0.0235 | 0.0033 | 1.10E-12 | rs7903146 | T | C | 0.25 | 50.71166208 |
| BMI | 0.0379 | 0.0067 | 1.27E-08 | rs7899106 | G | A | 0.05 | 31.99844063 |
| BMI | 0.0249 | 0.0037 | 2.19E-11 | rs17094222 | C | T | 0.2083 | 45.28926224 |
| BMI | -0.0185 | 0.0033 | 3.47E-08 | rs2176598 | C | T | 0.8 | 31.42791552 |
| BMI | 0.0256 | 0.003 | 1.17E-17 | rs3817334 | T | C | 0.45 | 72.81777778 |
| BMI | 0.0211 | 0.0029 | 5.44E-13 | rs12286929 | G | A | 0.4333 | 52.93816885 |
| BMI | 0.0206 | 0.003 | 6.67E-12 | rs10840100 | G | A | 0.725 | 47.15111111 |
| BMI | -0.0416 | 0.0037 | 6.66E-30 | rs11030104 | G | A | 0.2 | 126.4105186 |
| BMI | 0.032 | 0.003 | 5.11E-26 | rs7138803 | A | G | 0.4417 | 113.7777778 |
| BMI | -0.0304 | 0.0053 | 1.22E-08 | rs11057405 | A | G | 0.0917 | 32.8999644 |
| BMI | 0.0172 | 0.0031 | 2.96E-08 | rs1441264 | A | G | 0.55 | 30.78459938 |
| BMI | 0.0295 | 0.0046 | 1.43E-10 | rs9579083 | C | G | 0.2333 | 41.12712665 |
| BMI | 0.0324 | 0.0044 | 3.15E-13 | rs12429545 | A | G | 0.1 | 54.2231405 |
| BMI | -0.0182 | 0.0031 | 3.95E-09 | rs9540493 | G | A | 0.55 | 34.46826223 |
| BMI | 0.0274 | 0.0035 | 6.05E-15 | rs7144011 | T | G | 0.275 | 61.28653061 |
| BMI | -0.0221 | 0.0033 | 1.40E-11 | rs10132280 | A | C | 0.3333 | 44.84940312 |
| BMI | -0.0307 | 0.0035 | 1.53E-18 | rs13329567 | T | C | 0.2167 | 76.93795918 |
| BMI | -0.016 | 0.0029 | 4.52E-08 | rs3736485 | G | A | 0.575 | 30.43995244 |
| BMI | 0.0246 | 0.0037 | 3.90E-11 | rs12448257 | A | G | 0.225 | 44.20452885 |
| BMI | 0.0244 | 0.0039 | 3.94E-10 | rs879620 | T | C | 0.5917 | 39.1426693 |
| BMI | -0.0249 | 0.0038 | 8.55E-11 | rs9926784 | C | T | 0.2083 | 42.93698061 |
| BMI | -0.0187 | 0.003 | 6.58E-10 | rs4889606 | G | A | 0.3583 | 38.85444444 |
| BMI | 0.0803 | 0.003 | 2.17E-158 | rs1421085 | C | T | 0.45 | 716.4544444 |
| BMI | 0.0311 | 0.003 | 3.45E-25 | rs3888190 | A | C | 0.3583 | 107.4677778 |
| BMI | -0.0183 | 0.0029 | 3.64E-10 | rs12940622 | A | G | 0.4583 | 39.82045184 |
| BMI | 0.0184 | 0.0033 | 1.81E-08 | rs1000940 | G | A | 0.225 | 31.08907254 |
| BMI | 0.0562 | 0.0035 | 6.68E-59 | rs6567160 | C | T | 0.2833 | 257.8318367 |
| BMI | -0.0371 | 0.005 | 2.00E-13 | rs17066856 | C | T | 0.1333 | 55.0564 |
| BMI | 0.0209 | 0.0037 | 1.62E-08 | rs891389 | T | C | 0.325 | 31.90723156 |
| BMI | 0.0183 | 0.0033 | 1.92E-08 | rs14810 | G | C | 0.675 | 30.75206612 |
| BMI | -0.0339 | 0.0038 | 7.91E-19 | rs11672660 | T | C | 0.175 | 79.58518006 |
| BMI | 0.0243 | 0.0043 | 1.59E-08 | rs9304665 | A | T | 0.7 | 31.93564089 |
| BMI | -0.0196 | 0.0034 | 7.79E-09 | rs17724992 | G | A | 0.3083 | 33.23183391 |
| BMI | -0.0185 | 0.0033 | 2.14E-08 | rs6091540 | T | C | 0.275 | 31.42791552 |
| BMI | 0.0169 | 0.003 | 1.60E-08 | rs2836754 | C | T | 0.65 | 31.73444444 |
| VAT | 0.0125527 | 0.00196786 | 1.80E-10 | rs7537581 | A | C | 0.531743 | 40.6898326 |
| VAT | 0.0113099 | 0.00199809 | 1.50E-08 | rs4908672 | T | C | 0.392916 | 32.03962597 |
| VAT | -0.0147685 | 0.00228666 | 1.10E-10 | rs2791643 | T | C | 0.761787 | 41.71282323 |
| VAT | -0.0152607 | 0.00198063 | 1.30E-14 | rs12144626 | C | T | 0.582757 | 59.36660369 |
| VAT | 0.0127059 | 0.00207594 | 9.30E-10 | rs3766442 | C | T | 0.328381 | 37.46116434 |
| VAT | 0.020413 | 0.001989 | 1.00E-24 | rs2568958 | A | G | 0.603669 | 105.3280648 |
| VAT | 0.0174262 | 0.00233926 | 9.40E-14 | rs12072739 | G | A | 0.224583 | 55.49430271 |
| VAT | 0.0115824 | 0.00203229 | 1.20E-08 | rs1778830 | A | G | 0.362141 | 32.48072826 |
| VAT | -0.0436689 | 0.00591223 | 1.50E-13 | rs79518326 | A | C | 0.028638 | 54.55591739 |
| VAT | -0.0138291 | 0.00196314 | 1.90E-12 | rs815163 | C | T | 0.563206 | 49.62325978 |
| VAT | -0.0147572 | 0.00219669 | 1.80E-11 | rs10915840 | A | G | 0.274253 | 45.13052445 |
| VAT | -0.0158463 | 0.00278232 | 1.20E-08 | rs10927006 | C | T | 0.143706 | 32.43706807 |
| VAT | -0.0155056 | 0.00261946 | 3.20E-09 | rs4658403 | T | C | 0.833672 | 35.03915389 |
| VAT | 0.0190655 | 0.00258351 | 1.60E-13 | rs3766823 | A | G | 0.171973 | 54.45980786 |
| VAT | 0.0160792 | 0.00266375 | 1.60E-09 | rs61779305 | C | G | 0.172791 | 36.43694432 |
| VAT | -0.0178202 | 0.00241337 | 1.50E-13 | rs12724928 | C | T | 0.204845 | 54.52269673 |
| VAT | -0.0173264 | 0.00286467 | 1.50E-09 | rs4589116 | T | C | 0.865498 | 36.58200231 |
| VAT | 0.0444915 | 0.00241278 | 6.30E-76 | rs543874 | G | A | 0.205271 | 340.0311054 |
| VAT | -0.0153947 | 0.00240452 | 1.50E-10 | rs76702514 | G | C | 0.210651 | 40.99073189 |
| VAT | -0.0123954 | 0.00200114 | 5.90E-10 | rs11119208 | G | A | 0.614888 | 38.36773361 |
| VAT | 0.0227658 | 0.00215631 | 4.70E-26 | rs2494196 | A | C | 0.286018 | 111.4662623 |
| VAT | -0.018464 | 0.00261798 | 1.80E-12 | rs10799778 | G | T | 0.833665 | 49.74150646 |
| VAT | -0.0194482 | 0.00259868 | 7.20E-14 | rs3737992 | A | G | 0.168988 | 56.00840641 |
| VAT | -0.0188395 | 0.00209079 | 2.00E-19 | rs1167309 | T | C | 0.681108 | 81.19287435 |
| VAT | -0.0199173 | 0.00197478 | 6.40E-24 | rs1013293 | A | G | 0.43027 | 101.7240141 |
| VAT | 0.0142759 | 0.00195673 | 3.00E-13 | rs11208779 | C | G | 0.528904 | 53.2286176 |
| VAT | 0.0225892 | 0.00198498 | 5.30E-30 | rs11205303 | C | T | 0.406671 | 129.5058631 |
| VAT | 0.0140779 | 0.00236122 | 2.50E-09 | rs55816515 | C | T | 0.217763 | 35.54699111 |
| VAT | 0.0211528 | 0.00277793 | 2.60E-14 | rs61813293 | T | G | 0.14308 | 57.98199184 |
| VAT | -0.0171518 | 0.00225365 | 2.70E-14 | rs72634826 | A | G | 0.259876 | 57.9223894 |
| VAT | 0.0127606 | 0.00199634 | 1.60E-10 | rs3845344 | T | C | 0.391189 | 40.85763019 |
| VAT | 0.0199108 | 0.00197995 | 8.60E-24 | rs11165643 | T | C | 0.590189 | 101.1274308 |
| VAT | 0.0665947 | 0.00615325 | 2.70E-27 | rs17024393 | C | T | 0.02591 | 117.1305582 |
| VAT | 0.0110373 | 0.0019837 | 2.60E-08 | rs17781552 | A | G | 0.545262 | 30.95805785 |
| VAT | 0.0142452 | 0.00215289 | 3.70E-11 | rs6684205 | G | A | 0.286862 | 43.78178068 |
| VAT | -0.0135032 | 0.00214264 | 2.90E-10 | rs12031634 | A | G | 0.296495 | 39.71686711 |
| VAT | 0.0432047 | 0.00301618 | 1.50E-46 | rs34517439 | A | C | 0.121701 | 205.1858823 |
| VAT | 0.012894 | 0.00212701 | 1.30E-09 | rs6688826 | C | T | 0.298242 | 36.74821667 |
| VAT | 0.0124378 | 0.00200831 | 5.90E-10 | rs197422 | A | C | 0.379823 | 38.35532231 |
| VAT | 0.0211458 | 0.00205731 | 8.80E-25 | rs2678204 | G | T | 0.340185 | 105.644956 |
| VAT | 0.0240987 | 0.00194906 | 4.10E-35 | rs6752378 | A | C | 0.486283 | 152.8751208 |
| VAT | -0.0135316 | 0.00195977 | 5.00E-12 | rs12475388 | A | G | 0.485684 | 47.67471355 |
| VAT | -0.0140538 | 0.00234362 | 2.00E-09 | rs113019802 | A | G | 0.22505 | 35.95945877 |
| VAT | -0.013662 | 0.00202539 | 1.50E-11 | rs10187101 | T | C | 0.363607 | 45.4999835 |
| VAT | -0.0194111 | 0.00196687 | 5.70E-23 | rs1861410 | T | C | 0.555339 | 97.39776281 |
| VAT | 0.0114546 | 0.00204093 | 2.00E-08 | rs2692741 | C | G | 0.367191 | 31.49949832 |
| VAT | -0.0148033 | 0.0022222 | 2.70E-11 | rs1446585 | G | A | 0.244552 | 44.37626993 |
| VAT | 0.0182985 | 0.00320833 | 1.20E-08 | rs13409967 | T | G | 0.103039 | 32.52916506 |
| VAT | 0.01447 | 0.00201321 | 6.60E-13 | rs12987931 | T | C | 0.614937 | 51.66053558 |
| VAT | 0.0167032 | 0.00206274 | 5.60E-16 | rs2216931 | A | C | 0.66198 | 65.57078483 |
| VAT | -0.0783974 | 0.00455712 | 2.50E-66 | rs62107261 | C | T | 0.048321 | 295.952757 |
| VAT | -0.0210772 | 0.0019987 | 5.30E-26 | rs7575523 | G | T | 0.603215 | 111.2066116 |
| VAT | -0.0151584 | 0.00197504 | 1.70E-14 | rs429343 | G | A | 0.576574 | 58.9053763 |
| VAT | -0.0134785 | 0.00221652 | 1.20E-09 | rs13427822 | G | A | 0.271181 | 36.9776934 |
| VAT | -0.0132016 | 0.00207426 | 2.00E-10 | rs2943634 | C | A | 0.672555 | 40.50668984 |
| VAT | 0.0154229 | 0.00205116 | 5.50E-14 | rs10209821 | T | C | 0.342644 | 56.53703229 |
| VAT | 0.0145785 | 0.00254987 | 1.10E-08 | rs6735393 | C | A | 0.180527 | 32.68809441 |
| VAT | -0.0133694 | 0.00216591 | 6.70E-10 | rs396354 | C | T | 0.715675 | 38.10158316 |
| VAT | 0.0165 | 0.00199334 | 1.30E-16 | rs13389219 | T | C | 0.392426 | 68.51807056 |
| VAT | 0.0107453 | 0.00195816 | 4.10E-08 | rs55685371 | A | T | 0.553532 | 30.11207896 |
| VAT | -0.0299133 | 0.00366606 | 3.40E-16 | rs4482463 | A | C | 0.923039 | 66.57781163 |
| VAT | 0.0157296 | 0.00279234 | 1.80E-08 | rs1263629 | G | A | 0.143757 | 31.73209621 |
| VAT | 0.0471154 | 0.00258261 | 2.30E-74 | rs6744646 | G | A | 0.828314 | 332.8189978 |
| VAT | 0.018455 | 0.00334767 | 3.50E-08 | rs62124717 | A | G | 0.0947 | 30.39084799 |
| VAT | -0.0185222 | 0.00198725 | 1.20E-20 | rs1160543 | T | C | 0.400832 | 86.87206146 |
| VAT | -0.0145805 | 0.00250456 | 5.80E-09 | rs13008033 | G | A | 0.185807 | 33.89081041 |
| VAT | -0.0118348 | 0.00196344 | 1.70E-09 | rs11677541 | A | G | 0.535228 | 36.33177177 |
| VAT | -0.0180733 | 0.00251355 | 6.50E-13 | rs72917533 | C | T | 0.185501 | 51.70110885 |
| VAT | -0.0142962 | 0.00232419 | 7.70E-10 | rs79847714 | C | T | 0.228218 | 37.835369 |
| VAT | 0.0186983 | 0.00209543 | 4.50E-19 | rs35882248 | T | C | 0.317111 | 79.62655601 |
| VAT | -0.0108669 | 0.00196514 | 3.20E-08 | rs1840126 | C | A | 0.563615 | 30.5790754 |
| VAT | 0.0155506 | 0.00271607 | 1.00E-08 | rs111942358 | C | G | 0.153365 | 32.78025922 |
| VAT | 0.0180564 | 0.00201927 | 3.80E-19 | rs10175266 | G | A | 0.368857 | 79.96014038 |
| VAT | -0.0157786 | 0.00197847 | 1.50E-15 | rs13408397 | T | C | 0.411036 | 63.60305767 |
| VAT | 0.0142917 | 0.00195402 | 2.60E-13 | rs441792 | G | A | 0.486643 | 53.49457893 |
| VAT | -0.0136014 | 0.00223515 | 1.20E-09 | rs3754963 | T | A | 0.255045 | 37.03001416 |
| VAT | 0.0126522 | 0.00211613 | 2.20E-09 | rs73985439 | C | A | 0.307339 | 35.7476427 |
| VAT | -0.0138327 | 0.00195785 | 1.60E-12 | rs1554654 | T | C | 0.470556 | 49.91775964 |
| VAT | 0.0146472 | 0.00207288 | 1.60E-12 | rs9968060 | T | C | 0.642602 | 49.92992351 |
| VAT | -0.0120287 | 0.00206495 | 5.70E-09 | rs6769617 | T | A | 0.663975 | 33.93269133 |
| VAT | 0.0224847 | 0.00305205 | 1.70E-13 | rs9851777 | C | T | 0.884948 | 54.27388456 |
| VAT | 0.0126693 | 0.00212637 | 2.60E-09 | rs1078455 | C | T | 0.309538 | 35.49993578 |
| VAT | 0.0214248 | 0.00322004 | 2.90E-11 | rs3774063 | T | C | 0.102135 | 44.27015267 |
| VAT | 0.0110499 | 0.00199389 | 3.00E-08 | rs11720292 | A | C | 0.45852 | 30.71243886 |
| VAT | 0.0143852 | 0.00197796 | 3.50E-13 | rs1436348 | G | A | 0.582731 | 52.89282939 |
| VAT | 0.0206294 | 0.00218265 | 3.30E-21 | rs11709402 | G | A | 0.278651 | 89.33157563 |
| VAT | 0.0112074 | 0.0019503 | 9.10E-09 | rs6790206 | G | A | 0.541051 | 33.02226844 |
| VAT | 0.0146589 | 0.00203437 | 5.80E-13 | rs7372674 | A | C | 0.357186 | 51.92097971 |
| VAT | 0.0137529 | 0.00198694 | 4.50E-12 | rs7619139 | A | T | 0.588629 | 47.90921607 |
| VAT | -0.0187817 | 0.00255183 | 1.80E-13 | rs28350 | G | A | 0.820601 | 54.17093366 |
| VAT | 0.0248378 | 0.00195189 | 4.30E-37 | rs9843653 | C | T | 0.511529 | 161.9256217 |
| VAT | -0.0215606 | 0.00276035 | 5.70E-15 | rs17668356 | G | C | 0.145987 | 61.00892612 |
| VAT | -0.0124039 | 0.00221031 | 2.00E-08 | rs11926024 | A | T | 0.263929 | 31.49271736 |
| VAT | 0.0311211 | 0.00299903 | 3.20E-25 | rs1801282 | G | C | 0.119546 | 107.6832756 |
| VAT | 0.0124127 | 0.00196588 | 2.70E-10 | rs1857883 | A | G | 0.545009 | 39.86745471 |
| VAT | 0.0213268 | 0.00307284 | 3.90E-12 | rs754635 | G | C | 0.886587 | 48.16942891 |
| VAT | 0.0116765 | 0.00201566 | 6.90E-09 | rs3772882 | A | C | 0.373259 | 33.55759376 |
| VAT | -0.0162889 | 0.00201752 | 6.80E-16 | rs9876664 | T | G | 0.375486 | 65.18502207 |
| VAT | -0.0192152 | 0.00195062 | 6.80E-23 | rs1454687 | G | C | 0.51535 | 97.03858894 |
| VAT | 0.0225939 | 0.00196048 | 9.90E-31 | rs7632381 | C | T | 0.444374 | 132.8181945 |
| VAT | -0.0112827 | 0.0020317 | 2.80E-08 | rs361222 | T | A | 0.632467 | 30.83947102 |
| VAT | -0.0133068 | 0.00217018 | 8.70E-10 | rs11129446 | A | T | 0.285782 | 37.59722102 |
| VAT | -0.0118429 | 0.00200535 | 3.50E-09 | rs6782714 | T | C | 0.409425 | 34.87673003 |
| VAT | 0.0109217 | 0.00199148 | 4.20E-08 | rs4123668 | C | T | 0.402759 | 30.07658945 |
| VAT | -0.0144833 | 0.00211554 | 7.60E-12 | rs9839081 | A | G | 0.325039 | 46.86974283 |
| VAT | 0.0131288 | 0.00215496 | 1.10E-09 | rs1964675 | T | C | 0.711071 | 37.11689501 |
| VAT | 0.015542 | 0.00284839 | 4.90E-08 | rs12107172 | G | A | 0.136512 | 29.77247261 |
| VAT | 0.0171575 | 0.002003 | 1.10E-17 | rs1568488 | C | G | 0.594862 | 73.37466248 |
| VAT | 0.0160216 | 0.00214912 | 9.00E-14 | rs8192675 | C | T | 0.28869 | 55.57640338 |
| VAT | -0.0133717 | 0.0020414 | 5.70E-11 | rs2606227 | C | T | 0.631414 | 42.90590116 |
| VAT | 0.0180427 | 0.00312853 | 8.10E-09 | rs73175572 | G | A | 0.111713 | 33.26001252 |
| VAT | -0.0211658 | 0.00251576 | 4.00E-17 | rs73052033 | C | T | 0.185065 | 70.78332522 |
| VAT | -0.0158015 | 0.00287282 | 3.80E-08 | rs2269487 | G | A | 0.134728 | 30.25379082 |
| VAT | 0.0186849 | 0.00195803 | 1.40E-21 | rs2192527 | G | A | 0.465514 | 91.06319268 |
| VAT | -0.0173443 | 0.00235571 | 1.80E-13 | rs56203712 | G | A | 0.233836 | 54.2088136 |
| VAT | -0.0230745 | 0.00280342 | 1.90E-16 | rs73213484 | T | A | 0.141217 | 67.74671846 |
| VAT | 0.0139501 | 0.00225241 | 5.90E-10 | rs62302286 | C | T | 0.251284 | 38.35833503 |
| VAT | -0.0166952 | 0.00197551 | 2.90E-17 | rs1296328 | C | A | 0.558984 | 71.42081262 |
| VAT | -0.0365007 | 0.00624845 | 5.20E-09 | rs148636479 | T | A | 0.028546 | 34.12383149 |
| VAT | 0.0111139 | 0.00195256 | 1.30E-08 | rs11731255 | G | A | 0.473985 | 32.39844702 |
| VAT | 0.0141758 | 0.00195841 | 4.50E-13 | rs6840236 | C | T | 0.464888 | 52.39476762 |
| VAT | 0.0125242 | 0.00198677 | 2.90E-10 | rs4419475 | T | A | 0.407357 | 39.73788983 |
| VAT | 0.0420959 | 0.00592358 | 1.20E-12 | rs1229984 | C | T | 0.972782 | 50.50229121 |
| VAT | 0.0375077 | 0.00371154 | 5.20E-24 | rs13107325 | T | C | 0.074872 | 102.1251192 |
| VAT | -0.013819 | 0.00203762 | 1.20E-11 | rs11099020 | T | C | 0.640531 | 45.99459978 |
| VAT | -0.0163404 | 0.00196127 | 8.00E-17 | rs4240326 | G | A | 0.550137 | 69.4145634 |
| VAT | -0.0138687 | 0.00248678 | 2.40E-08 | rs113079574 | T | C | 0.192709 | 31.10260579 |
| VAT | -0.0109141 | 0.00194867 | 2.10E-08 | rs7663885 | C | T | 0.500214 | 31.36889778 |
| VAT | 0.0112973 | 0.00198568 | 1.30E-08 | rs530255 | C | T | 0.411125 | 32.3691131 |
| VAT | -0.0162395 | 0.00220477 | 1.80E-13 | rs3113509 | T | C | 0.731949 | 54.2523713 |
| VAT | -0.0107953 | 0.00197091 | 4.30E-08 | rs7683836 | A | G | 0.557258 | 30.00100792 |
| VAT | 0.011656 | 0.00205975 | 1.50E-08 | rs4088476 | T | C | 0.660801 | 32.02359277 |
| VAT | 0.013355 | 0.00243296 | 4.00E-08 | rs35568752 | A | G | 0.201678 | 30.13129845 |
| VAT | 0.0275414 | 0.00197323 | 2.80E-44 | rs10938398 | A | G | 0.433522 | 194.8124043 |
| VAT | -0.0118394 | 0.00197949 | 2.20E-09 | rs2237025 | C | T | 0.555383 | 35.77278588 |
| VAT | -0.0131276 | 0.0023739 | 3.20E-08 | rs1383723 | T | A | 0.78314 | 30.58058785 |
| VAT | 0.01403 | 0.0020466 | 7.10E-12 | rs6847975 | A | G | 0.356172 | 46.99475637 |
| VAT | -0.0121038 | 0.0019543 | 5.90E-10 | rs1350506 | C | T | 0.47058 | 38.35844682 |
| VAT | -0.0330784 | 0.00486322 | 1.00E-11 | rs72767253 | A | C | 0.042122 | 46.2637845 |
| VAT | 0.0112111 | 0.00196444 | 1.10E-08 | rs467176 | T | C | 0.545076 | 32.57008676 |
| VAT | -0.0138893 | 0.00228563 | 1.20E-09 | rs10071662 | T | C | 0.242255 | 36.92741112 |
| VAT | -0.0140621 | 0.00211678 | 3.10E-11 | rs2964023 | C | T | 0.691967 | 44.13152316 |
| VAT | 0.0139637 | 0.00195362 | 8.80E-13 | rs1503526 | C | T | 0.480033 | 51.08822738 |
| VAT | -0.0123008 | 0.00208579 | 3.70E-09 | rs10050620 | T | C | 0.325381 | 34.77967767 |
| VAT | 0.0146571 | 0.00208292 | 2.00E-12 | rs11135450 | G | A | 0.668911 | 49.51661197 |
| VAT | -0.0127468 | 0.00197561 | 1.10E-10 | rs396755 | G | C | 0.571066 | 41.62937703 |
| VAT | 0.0161316 | 0.00202598 | 1.70E-15 | rs3822742 | A | C | 0.371036 | 63.39931737 |
| VAT | 0.0176994 | 0.00219661 | 7.80E-16 | rs812949 | C | T | 0.729231 | 64.92488301 |
| VAT | 0.0179075 | 0.00291413 | 8.00E-10 | rs9654453 | C | T | 0.12928 | 37.76174681 |
| VAT | -0.0125974 | 0.00198674 | 2.30E-10 | rs2610245 | G | A | 0.517849 | 40.20497234 |
| VAT | -0.0185586 | 0.0020343 | 7.30E-20 | rs7707394 | A | G | 0.357298 | 83.22626875 |
| VAT | -0.0179935 | 0.00226342 | 1.90E-15 | rs252749 | A | G | 0.245964 | 63.19766003 |
| VAT | 0.0324202 | 0.00286872 | 1.30E-29 | rs34483452 | A | C | 0.136334 | 127.7188669 |
| VAT | -0.0198339 | 0.00255126 | 7.60E-15 | rs40071 | C | T | 0.179506 | 60.43754273 |
| VAT | -0.0122788 | 0.00196155 | 3.90E-10 | rs698147 | G | A | 0.543491 | 39.18438956 |
| VAT | -0.0265622 | 0.00199805 | 2.50E-40 | rs2307111 | C | T | 0.395076 | 176.7320768 |
| VAT | 0.0129733 | 0.00224132 | 7.10E-09 | rs159037 | C | T | 0.253896 | 33.50373223 |
| VAT | 0.0111105 | 0.00198188 | 2.10E-08 | rs62379271 | G | T | 0.578512 | 31.42769266 |
| VAT | 0.0132856 | 0.00197516 | 1.70E-11 | rs10478110 | C | A | 0.434339 | 45.24366536 |
| VAT | 0.013152 | 0.0019868 | 3.60E-11 | rs347551 | G | C | 0.472292 | 43.82029508 |
| VAT | -0.0144847 | 0.00197176 | 2.00E-13 | rs4958702 | C | T | 0.572323 | 53.96484144 |
| VAT | 0.0382694 | 0.00551616 | 4.00E-12 | rs75281888 | C | T | 0.032469 | 48.13152224 |
| VAT | -0.0211336 | 0.00238351 | 7.50E-19 | rs7442885 | G | C | 0.214107 | 78.6163741 |
| VAT | -0.0160129 | 0.0023545 | 1.00E-11 | rs1363695 | T | C | 0.225976 | 46.25328615 |
| VAT | -0.0166619 | 0.00292777 | 1.30E-08 | rs253440 | T | G | 0.127882 | 32.38732911 |
| VAT | 0.0115143 | 0.00197884 | 5.90E-09 | rs13354321 | C | T | 0.409862 | 33.85740902 |
| VAT | 0.0308412 | 0.00442333 | 3.10E-12 | rs41271299 | T | C | 0.051245 | 48.6142795 |
| VAT | 0.0193946 | 0.00209518 | 2.10E-20 | rs17681686 | C | G | 0.304859 | 85.68779641 |
| VAT | 0.034486 | 0.00258879 | 1.70E-40 | rs72892910 | T | G | 0.172203 | 177.4565374 |
| VAT | 0.0142319 | 0.00204964 | 3.80E-12 | rs2499468 | A | C | 0.650983 | 48.21371441 |
| VAT | 0.0148707 | 0.00260229 | 1.10E-08 | rs1031881 | G | T | 0.169253 | 32.65513165 |
| VAT | -0.0178981 | 0.00228474 | 4.70E-15 | rs6569648 | T | C | 0.760889 | 61.36776267 |
| VAT | -0.0138972 | 0.00195198 | 1.10E-12 | rs765874 | A | T | 0.489318 | 50.68785208 |
| VAT | 0.0152667 | 0.00245891 | 5.30E-10 | rs9378684 | T | C | 0.200586 | 38.54828638 |
| VAT | -0.0161903 | 0.00205102 | 2.90E-15 | rs4377779 | C | T | 0.346841 | 62.31175764 |
| VAT | -0.0351601 | 0.0022264 | 3.50E-56 | rs62396185 | C | G | 0.259954 | 249.3984882 |
| VAT | 0.0150394 | 0.00227821 | 4.10E-11 | rs62405861 | A | C | 0.241465 | 43.57862398 |
| VAT | -0.0125138 | 0.00195609 | 1.60E-10 | rs998584 | A | C | 0.48286 | 40.92613699 |
| VAT | 0.0179661 | 0.00298029 | 1.70E-09 | rs13191298 | G | A | 0.122241 | 36.34047287 |
| VAT | -0.0239116 | 0.00305945 | 5.50E-15 | rs314279 | A | C | 0.884507 | 61.08443401 |
| VAT | 0.020442 | 0.00201791 | 4.10E-24 | rs2802295 | G | A | 0.626185 | 102.62264 |
| VAT | 0.0125735 | 0.00215952 | 5.80E-09 | rs7762794 | G | A | 0.285529 | 33.89986051 |
| VAT | -0.0258212 | 0.00289453 | 4.60E-19 | rs28366156 | C | T | 0.130599 | 79.57868033 |
| VAT | 0.044939 | 0.00280896 | 1.30E-57 | rs2814993 | A | G | 0.139796 | 255.9503328 |
| VAT | 0.0118428 | 0.00216957 | 4.80E-08 | rs12528644 | A | C | 0.283817 | 29.79623948 |
| VAT | 0.0137122 | 0.00196418 | 2.90E-12 | rs9294260 | A | G | 0.476616 | 48.73620506 |
| VAT | -0.013859 | 0.00221512 | 3.90E-10 | rs10499014 | G | C | 0.26865 | 39.14436686 |
| VAT | 0.0188041 | 0.00199609 | 4.50E-21 | rs9320823 | C | T | 0.602914 | 88.74519874 |
| VAT | 0.0118063 | 0.00196914 | 2.00E-09 | rs3853252 | A | G | 0.454896 | 35.94797579 |
| VAT | -0.0132445 | 0.00222371 | 2.60E-09 | rs854917 | T | C | 0.735788 | 35.4743819 |
| VAT | -0.0149169 | 0.00206744 | 5.40E-13 | rs13218383 | G | C | 0.33511 | 52.0584613 |
| VAT | 0.0131824 | 0.00195149 | 1.40E-11 | rs1159974 | C | T | 0.524326 | 45.6306138 |
| VAT | 0.0138387 | 0.00211712 | 6.30E-11 | rs4709745 | C | T | 0.307212 | 42.72672836 |
| VAT | -0.0162386 | 0.0020116 | 6.90E-16 | rs215634 | G | A | 0.611924 | 65.16492719 |
| VAT | -0.012672 | 0.00197257 | 1.30E-10 | rs3807566 | T | G | 0.438317 | 41.2691459 |
| VAT | 0.0126406 | 0.00199534 | 2.40E-10 | rs6973656 | G | A | 0.397015 | 40.13299396 |
| VAT | 0.015535 | 0.00202571 | 1.70E-14 | rs10248298 | A | C | 0.365966 | 58.8122739 |
| VAT | -0.0140409 | 0.00241705 | 6.30E-09 | rs6973700 | G | A | 0.206152 | 33.74571417 |
| VAT | 0.0146651 | 0.0019903 | 1.70E-13 | rs4718964 | T | G | 0.413152 | 54.29164135 |
| VAT | -0.015801 | 0.00222183 | 1.10E-12 | rs12538435 | G | A | 0.26181 | 50.57635108 |
| VAT | 0.0115705 | 0.00196603 | 4.00E-09 | rs2470937 | A | T | 0.451438 | 34.63570024 |
| VAT | 0.013258 | 0.00202448 | 5.80E-11 | rs6946091 | G | A | 0.627423 | 42.88733379 |
| VAT | -0.0203673 | 0.00212386 | 8.80E-22 | rs1182199 | A | C | 0.304437 | 91.96342756 |
| VAT | 0.0200218 | 0.00288457 | 3.90E-12 | rs35589149 | C | G | 0.132426 | 48.17747922 |
| VAT | -0.0172071 | 0.00276169 | 4.60E-10 | rs17704028 | T | C | 0.147185 | 38.82089204 |
| VAT | -0.0172661 | 0.00198979 | 4.10E-18 | rs2396625 | A | T | 0.421312 | 75.2963659 |
| VAT | 0.0109837 | 0.00196568 | 2.30E-08 | rs2192649 | G | T | 0.500768 | 31.22278849 |
| VAT | 0.0155148 | 0.00206143 | 5.20E-14 | rs60814640 | G | A | 0.345365 | 56.64416497 |
| VAT | 0.0124526 | 0.00195649 | 2.00E-10 | rs35722851 | C | T | 0.491438 | 40.51023952 |
| VAT | -0.0193703 | 0.00292146 | 3.30E-11 | rs6461999 | A | C | 0.870776 | 43.96153063 |
| VAT | -0.0107742 | 0.00196831 | 4.40E-08 | rs62443626 | A | G | 0.463938 | 29.96284642 |
| VAT | -0.01292 | 0.00200597 | 1.20E-10 | rs2289379 | T | C | 0.39566 | 41.48357344 |
| VAT | -0.0174463 | 0.00198456 | 1.50E-18 | rs58862095 | T | C | 0.419231 | 77.28197371 |
| VAT | -0.0167278 | 0.0026847 | 4.60E-10 | rs10257197 | G | A | 0.841645 | 38.82273514 |
| VAT | 0.0160822 | 0.0028894 | 2.60E-08 | rs142583374 | A | G | 0.132544 | 30.97958392 |
| VAT | 0.0128414 | 0.00221452 | 6.70E-09 | rs13252030 | G | A | 0.732639 | 33.62525097 |
| VAT | 0.0126391 | 0.00196524 | 1.30E-10 | rs10099330 | G | A | 0.45291 | 41.36195995 |
| VAT | 0.0114526 | 0.00197487 | 6.70E-09 | rs7460093 | A | G | 0.53113 | 33.63033241 |
| VAT | -0.0228497 | 0.00216201 | 4.20E-26 | rs7845090 | A | G | 0.70896 | 111.6980543 |
| VAT | 0.0111885 | 0.00199878 | 2.20E-08 | rs2920939 | A | G | 0.606689 | 31.3338487 |
| VAT | 0.0226808 | 0.00217833 | 2.20E-25 | rs4876611 | G | A | 0.720231 | 108.4100121 |
| VAT | 0.0171594 | 0.00312884 | 4.20E-08 | rs7828631 | T | C | 0.109944 | 30.0772057 |
| VAT | -0.0162132 | 0.00297266 | 4.90E-08 | rs34571768 | A | G | 0.126421 | 29.7472622 |
| VAT | -0.0121079 | 0.00196073 | 6.60E-10 | rs1216547 | T | A | 0.476811 | 38.1330957 |
| VAT | 0.0130436 | 0.00216401 | 1.70E-09 | rs1813039 | A | G | 0.709735 | 36.33092184 |
| VAT | 0.0194145 | 0.00197253 | 7.40E-23 | rs10100245 | A | G | 0.564528 | 96.87354359 |
| VAT | 0.014834 | 0.00203434 | 3.10E-13 | rs1106761 | A | G | 0.384047 | 53.17034429 |
| VAT | 0.0146922 | 0.00251611 | 5.20E-09 | rs11782341 | G | A | 0.188829 | 34.0968623 |
| VAT | 0.0225207 | 0.00331076 | 1.00E-11 | rs117176448 | G | C | 0.096193 | 46.27094526 |
| VAT | 0.0120742 | 0.00219168 | 3.60E-08 | rs6980476 | G | T | 0.272334 | 30.3502618 |
| VAT | -0.0128813 | 0.00235117 | 4.30E-08 | rs497905 | T | C | 0.221986 | 30.015897 |
| VAT | -0.0129104 | 0.00196549 | 5.10E-11 | rs11778934 | G | C | 0.536162 | 43.14571984 |
| VAT | -0.0126487 | 0.00197563 | 1.50E-10 | rs2717609 | T | A | 0.466095 | 40.9902493 |
| VAT | -0.0128485 | 0.00199312 | 1.10E-10 | rs6601527 | A | C | 0.589118 | 41.55640436 |
| VAT | 0.014401 | 0.00217517 | 3.60E-11 | rs62499697 | C | T | 0.29274 | 43.83276743 |
| VAT | -0.0118746 | 0.00200317 | 3.10E-09 | rs7823952 | T | G | 0.577984 | 35.14004905 |
| VAT | -0.0206159 | 0.00266577 | 1.00E-14 | rs11779446 | G | A | 0.161272 | 59.80799529 |
| VAT | 0.0158876 | 0.00195392 | 4.30E-16 | rs2954021 | G | A | 0.504587 | 66.1154621 |
| VAT | -0.0223076 | 0.00197517 | 1.40E-29 | rs13292699 | C | A | 0.433698 | 127.5547793 |
| VAT | -0.0179431 | 0.00304317 | 3.70E-09 | rs16916303 | G | A | 0.119756 | 34.76502349 |
| VAT | -0.0116902 | 0.00201834 | 7.00E-09 | rs7034554 | G | A | 0.373704 | 33.54711891 |
| VAT | -0.0143227 | 0.00206176 | 3.70E-12 | rs7038943 | C | T | 0.338801 | 48.25847252 |
| VAT | 0.0194261 | 0.00321643 | 1.50E-09 | rs7864914 | G | A | 0.103711 | 36.47732912 |
| VAT | -0.0112897 | 0.00201081 | 2.00E-08 | rs10959841 | C | T | 0.387368 | 31.52265076 |
| VAT | -0.0156105 | 0.00203905 | 1.90E-14 | rs10756798 | T | C | 0.642425 | 58.61083067 |
| VAT | -0.0173642 | 0.0029988 | 7.00E-09 | rs35307904 | A | G | 0.122351 | 33.5285332 |
| VAT | 0.012325 | 0.002137 | 8.00E-09 | rs17218712 | T | C | 0.304763 | 33.26326009 |
| VAT | 0.0130448 | 0.00196037 | 2.80E-11 | rs7357754 | G | A | 0.500141 | 44.27909688 |
| VAT | 0.0140728 | 0.00206238 | 8.90E-12 | rs7027304 | T | C | 0.652657 | 46.56114529 |
| VAT | 0.0117995 | 0.00207075 | 1.20E-08 | rs3739514 | A | G | 0.339665 | 32.469221 |
| VAT | -0.0248846 | 0.00406359 | 9.10E-10 | rs10116857 | A | C | 0.061829 | 37.50088894 |
| VAT | 0.0212749 | 0.00208654 | 2.10E-24 | rs17770336 | T | C | 0.322489 | 103.9636756 |
| VAT | -0.0139299 | 0.00218808 | 1.90E-10 | rs10820852 | A | C | 0.276324 | 40.5293468 |
| VAT | 0.0167542 | 0.00209908 | 1.40E-15 | rs2417084 | C | T | 0.315954 | 63.7073308 |
| VAT | -0.0112355 | 0.00204186 | 3.70E-08 | rs1147346 | G | A | 0.377895 | 30.27839744 |
| VAT | 0.0146196 | 0.00207681 | 1.90E-12 | rs7893571 | T | G | 0.665918 | 49.55385526 |
| VAT | 0.0125754 | 0.00212837 | 3.50E-09 | rs4919478 | G | A | 0.310977 | 34.90996059 |
| VAT | 0.0179707 | 0.00227166 | 2.60E-15 | rs10510025 | T | C | 0.247061 | 62.58111931 |
| VAT | 0.0151399 | 0.00217618 | 3.50E-12 | rs67609008 | C | T | 0.28361 | 48.4012284 |
| VAT | 0.0157879 | 0.00221376 | 9.90E-13 | rs10999456 | T | C | 0.267947 | 50.86132406 |
| VAT | 0.0138641 | 0.00196581 | 1.80E-12 | rs10887578 | C | G | 0.497547 | 49.73937049 |
| VAT | 0.0210978 | 0.00207656 | 3.00E-24 | rs11012732 | G | A | 0.331724 | 103.2251146 |
| VAT | 0.0129137 | 0.00226633 | 1.20E-08 | rs12762744 | T | C | 0.248356 | 32.46796661 |
| VAT | -0.0136583 | 0.00209269 | 6.70E-11 | rs12254441 | T | C | 0.37321 | 42.59743974 |
| VAT | -0.0219389 | 0.00297359 | 1.60E-13 | rs7916385 | T | C | 0.150254 | 54.43365821 |
| VAT | 0.0125829 | 0.00197795 | 2.00E-10 | rs11245344 | T | C | 0.571176 | 40.46978268 |
| VAT | -0.0147647 | 0.00198052 | 9.00E-14 | rs2172131 | C | T | 0.578558 | 55.57644833 |
| VAT | 0.0125945 | 0.00201698 | 4.30E-10 | rs743572 | G | A | 0.376538 | 38.99048863 |
| VAT | 0.0176652 | 0.00273642 | 1.10E-10 | rs16934748 | C | T | 0.150346 | 41.67461588 |
| VAT | -0.0122284 | 0.00209427 | 5.30E-09 | rs7070670 | T | C | 0.327958 | 34.09368385 |
| VAT | 0.0230515 | 0.00386381 | 2.40E-09 | rs17399739 | G | A | 0.068917 | 35.59318546 |
| VAT | 0.0186462 | 0.00196979 | 2.90E-21 | rs577525 | C | T | 0.562069 | 89.60676934 |
| VAT | -0.0111456 | 0.00202513 | 3.70E-08 | rs1008982 | C | T | 0.376587 | 30.29012677 |
| VAT | -0.0387799 | 0.00407213 | 1.70E-21 | rs35099456 | C | G | 0.062856 | 90.69223433 |
| VAT | 0.0156535 | 0.00224486 | 3.10E-12 | rs61903695 | G | A | 0.254882 | 48.6232957 |
| VAT | -0.0199119 | 0.00218994 | 9.70E-20 | rs885114 | A | G | 0.275571 | 82.67248132 |
| VAT | 0.0234274 | 0.00198294 | 3.30E-32 | rs7124681 | A | C | 0.408433 | 139.5818784 |
| VAT | -0.015254 | 0.00267467 | 1.20E-08 | rs72915955 | A | G | 0.161011 | 32.52573138 |
| VAT | 0.0112934 | 0.00195633 | 7.80E-09 | rs617948 | G | A | 0.519777 | 33.32461891 |
| VAT | 0.0501467 | 0.00802602 | 4.20E-10 | rs55707359 | G | T | 0.015442 | 39.03770179 |
| VAT | -0.0149119 | 0.00200551 | 1.00E-13 | rs719802 | C | T | 0.614396 | 55.28614413 |
| VAT | 0.0233786 | 0.004184 | 2.30E-08 | rs80197460 | G | C | 0.058184 | 31.22149171 |
| VAT | 0.0149978 | 0.00196395 | 2.20E-14 | rs7944782 | G | T | 0.509795 | 58.31687745 |
| VAT | 0.0175592 | 0.00263466 | 2.70E-11 | rs12364470 | G | T | 0.164515 | 44.41813248 |
| VAT | -0.0238076 | 0.00424847 | 2.10E-08 | rs1881505 | C | T | 0.943044 | 31.40263726 |
| VAT | 0.012072 | 0.00198918 | 1.30E-09 | rs1440286 | A | G | 0.412662 | 36.8307265 |
| VAT | 0.0176637 | 0.00223158 | 2.50E-15 | rs11030016 | T | C | 0.739778 | 62.6525049 |
| VAT | -0.0147924 | 0.00205311 | 5.80E-13 | rs1782508 | G | C | 0.655505 | 51.91021737 |
| VAT | -0.0189152 | 0.00286103 | 3.80E-11 | rs11603984 | T | G | 0.135128 | 43.70962212 |
| VAT | 0.0109701 | 0.00198315 | 3.20E-08 | rs553634 | A | C | 0.583605 | 30.59919804 |
| VAT | 0.0150043 | 0.00225978 | 3.10E-11 | rs1916039 | G | A | 0.252692 | 44.08584371 |
| VAT | 0.0118349 | 0.00196606 | 1.70E-09 | rs4936175 | C | T | 0.442747 | 36.23561615 |
| VAT | 0.013661 | 0.00198165 | 5.40E-12 | rs4757136 | A | T | 0.580291 | 47.52379124 |
| VAT | 0.0285045 | 0.00209435 | 3.50E-42 | rs1013402 | G | A | 0.318471 | 185.2372533 |
| VAT | 0.022946 | 0.00212771 | 4.10E-27 | rs59227842 | G | A | 0.311473 | 116.3025157 |
| VAT | 0.0189346 | 0.00263844 | 7.20E-13 | rs477895 | T | C | 0.836504 | 51.50125651 |
| VAT | 0.0137454 | 0.00247375 | 2.80E-08 | rs329651 | T | G | 0.804123 | 30.87472875 |
| VAT | 0.0165815 | 0.00283692 | 5.10E-09 | rs10505836 | C | A | 0.860026 | 34.16279952 |
| VAT | 0.0143399 | 0.00195592 | 2.30E-13 | rs1458156 | T | C | 0.488497 | 53.75143591 |
| VAT | -0.0380753 | 0.00570662 | 2.50E-11 | rs3730071 | A | C | 0.030238 | 44.51729418 |
| VAT | 0.0153517 | 0.00238412 | 1.20E-10 | rs7975187 | G | A | 0.21386 | 41.4626187 |
| VAT | -0.0140812 | 0.00203605 | 4.60E-12 | rs11105842 | A | G | 0.367545 | 47.83022855 |
| VAT | -0.011551 | 0.00199525 | 7.10E-09 | rs10783779 | G | T | 0.402975 | 33.5154094 |
| VAT | -0.0146733 | 0.00195894 | 6.90E-14 | rs10878349 | G | A | 0.513092 | 56.10651907 |
| VAT | 0.0170871 | 0.00197814 | 5.70E-18 | rs770082 | A | G | 0.436959 | 74.61440366 |
| VAT | 0.0160733 | 0.00199427 | 7.60E-16 | rs2287214 | G | A | 0.397779 | 64.95942754 |
| VAT | -0.0152505 | 0.00214522 | 1.20E-12 | rs12578258 | G | A | 0.290909 | 50.53874937 |
| VAT | -0.0430138 | 0.00354027 | 5.70E-34 | rs147730268 | T | G | 0.087269 | 147.6192019 |
| VAT | 0.0223062 | 0.00239713 | 1.30E-20 | rs55726687 | A | G | 0.209742 | 86.59005378 |
| VAT | 0.0669471 | 0.00757221 | 9.50E-19 | rs76895963 | G | T | 0.020665 | 78.16606306 |
| VAT | 0.0287176 | 0.00200949 | 2.50E-46 | rs7132908 | A | G | 0.384506 | 204.2323739 |
| VAT | -0.0250514 | 0.00427186 | 4.50E-09 | rs2731238 | T | G | 0.06751 | 34.38982615 |
| VAT | -0.015588 | 0.00209493 | 1.00E-13 | rs6606686 | C | G | 0.680575 | 55.36582328 |
| VAT | -0.025579 | 0.00440858 | 6.50E-09 | rs75412871 | T | C | 0.051924 | 33.66430645 |
| VAT | 0.0161498 | 0.00206722 | 5.60E-15 | rs12316080 | T | C | 0.335425 | 61.03246358 |
| VAT | 0.0121771 | 0.00208948 | 5.60E-09 | rs7960609 | G | A | 0.326898 | 33.96341157 |
| VAT | 0.0427975 | 0.00702405 | 1.10E-09 | rs61754230 | T | C | 0.019714 | 37.12458542 |
| VAT | -0.0184245 | 0.00292272 | 2.90E-10 | rs75524125 | A | T | 0.128491 | 39.73900592 |
| VAT | 0.0129143 | 0.00211789 | 1.10E-09 | rs900448 | G | C | 0.307474 | 37.18218798 |
| VAT | 0.0155474 | 0.00206908 | 5.70E-14 | rs1218822 | A | G | 0.661484 | 56.46261378 |
| VAT | 0.0121763 | 0.00197792 | 7.50E-10 | rs9533031 | T | G | 0.575427 | 37.89773334 |
| VAT | 0.0168227 | 0.00203425 | 1.30E-16 | rs1441264 | A | G | 0.593707 | 68.38844848 |
| VAT | 0.0123869 | 0.00197947 | 3.90E-10 | rs912690 | G | C | 0.449134 | 39.15862329 |
| VAT | 0.0144371 | 0.00208531 | 4.40E-12 | rs1336486 | G | T | 0.328658 | 47.93124144 |
| VAT | -0.0134337 | 0.00227678 | 3.60E-09 | rs6561937 | A | T | 0.753678 | 34.81361766 |
| VAT | 0.0152468 | 0.00219909 | 4.10E-12 | rs61969510 | C | T | 0.279057 | 48.06969866 |
| VAT | -0.014868 | 0.00215462 | 5.20E-12 | rs12869627 | A | G | 0.28901 | 47.6171865 |
| VAT | 0.0115249 | 0.00199486 | 7.60E-09 | rs9888533 | T | C | 0.538126 | 33.37716819 |
| VAT | -0.0180893 | 0.00245572 | 1.80E-13 | rs9316661 | C | T | 0.801418 | 54.26075448 |
| VAT | -0.0127095 | 0.00221203 | 9.20E-09 | rs2249825 | C | G | 0.266655 | 33.0122333 |
| VAT | 0.029284 | 0.00294554 | 2.70E-23 | rs4477562 | T | C | 0.128613 | 98.83959264 |
| VAT | -0.0175549 | 0.00253518 | 4.40E-12 | rs11839227 | C | T | 0.183332 | 47.94895203 |
| VAT | -0.0149957 | 0.00198423 | 4.10E-14 | rs9522183 | T | G | 0.561749 | 57.11490567 |
| VAT | 0.0134123 | 0.00199645 | 1.80E-11 | rs9515455 | A | G | 0.414845 | 45.13252609 |
| VAT | 0.0136413 | 0.00214721 | 2.10E-10 | rs10141106 | G | A | 0.703646 | 40.36105204 |
| VAT | 0.0266959 | 0.00235343 | 8.00E-30 | rs10146997 | G | A | 0.221894 | 128.6727442 |
| VAT | -0.0123581 | 0.00221021 | 2.30E-08 | rs10132514 | T | C | 0.273267 | 31.26340893 |
| VAT | -0.013743 | 0.00224905 | 9.90E-10 | rs2103785 | T | C | 0.255982 | 37.33918821 |
| VAT | -0.0221218 | 0.00227487 | 2.40E-22 | rs9788550 | C | G | 0.247503 | 94.5644185 |
| VAT | -0.0173799 | 0.00197442 | 1.30E-18 | rs2143975 | G | C | 0.533031 | 77.48461213 |
| VAT | -0.0133672 | 0.00195882 | 8.80E-12 | rs12885458 | G | T | 0.507926 | 46.56845534 |
| VAT | -0.0163725 | 0.00207372 | 2.90E-15 | rs3803286 | G | A | 0.666738 | 62.3346845 |
| VAT | 0.0169888 | 0.002204 | 1.30E-14 | rs217672 | C | A | 0.271743 | 59.41583802 |
| VAT | 0.0226319 | 0.00354724 | 1.80E-10 | rs12881629 | G | A | 0.082658 | 40.70623026 |
| VAT | -0.0148335 | 0.00245344 | 1.50E-09 | rs4430672 | C | T | 0.800889 | 36.55412467 |
| VAT | 0.0140913 | 0.00204721 | 5.90E-12 | rs12890931 | G | T | 0.36239 | 47.37806653 |
| VAT | 0.0150961 | 0.00200542 | 5.20E-14 | rs10144067 | T | C | 0.591293 | 56.66551555 |
| VAT | 0.0183932 | 0.00203768 | 1.80E-19 | rs6575340 | A | G | 0.636047 | 81.47842413 |
| VAT | -0.0145622 | 0.00204493 | 1.10E-12 | rs61992671 | G | A | 0.492084 | 50.71040632 |
| VAT | 0.0230668 | 0.00355518 | 8.70E-11 | rs79197301 | A | T | 0.082564 | 42.09703534 |
| VAT | -0.0266406 | 0.00233792 | 4.40E-30 | rs28587941 | C | T | 0.224977 | 129.8460406 |
| VAT | 0.0250376 | 0.00347887 | 6.20E-13 | rs35697691 | G | C | 0.089384 | 51.79752319 |
| VAT | 0.0139553 | 0.00222748 | 3.70E-10 | rs2460 | A | G | 0.262582 | 39.25100016 |
| VAT | -0.0385285 | 0.00667383 | 7.80E-09 | rs8026411 | T | C | 0.022436 | 33.3283584 |
| VAT | 0.0175619 | 0.00207457 | 2.60E-17 | rs7171864 | A | G | 0.660091 | 71.66165158 |
| VAT | 0.0125923 | 0.00205198 | 8.40E-10 | rs7167767 | A | G | 0.651966 | 37.6585745 |
| VAT | -0.0252975 | 0.00218025 | 4.00E-31 | rs62025831 | C | T | 0.28054 | 134.6302396 |
| VAT | -0.0160124 | 0.00271039 | 3.50E-09 | rs1874832 | A | G | 0.84555 | 34.90191756 |
| VAT | -0.013424 | 0.00221293 | 1.30E-09 | rs11856579 | A | G | 0.267333 | 36.79836596 |
| VAT | -0.0158556 | 0.00220564 | 6.50E-13 | rs11854132 | A | G | 0.270185 | 51.67685839 |
| VAT | -0.0162331 | 0.00221707 | 2.40E-13 | rs3817428 | G | C | 0.264989 | 53.60979139 |
| VAT | -0.0230331 | 0.00310531 | 1.20E-13 | rs72755233 | A | G | 0.111427 | 55.01674123 |
| VAT | 0.0241441 | 0.00201244 | 3.70E-33 | rs879620 | T | C | 0.613268 | 143.9382309 |
| VAT | 0.0311398 | 0.00199676 | 7.90E-55 | rs7498665 | G | A | 0.399561 | 243.2091454 |
| VAT | -0.0149117 | 0.0024038 | 5.50E-10 | rs2966859 | G | A | 0.789 | 38.48200165 |
| VAT | -0.0215622 | 0.00254086 | 2.10E-17 | rs13333747 | C | T | 0.18275 | 72.01528229 |
| VAT | 0.0225304 | 0.00196129 | 1.50E-30 | rs3814883 | T | C | 0.482265 | 131.9636066 |
| VAT | 0.0625602 | 0.0019907 | 1.00E-200 | rs56094641 | G | A | 0.404637 | 987.6080564 |
| VAT | -0.0130412 | 0.00201989 | 1.10E-10 | rs756717 | A | G | 0.398954 | 41.68498719 |
| VAT | -0.0198113 | 0.00294036 | 1.60E-11 | rs67689854 | A | C | 0.129883 | 45.39676824 |
| VAT | -0.0129567 | 0.0021449 | 1.50E-09 | rs11075256 | C | G | 0.703471 | 36.49007097 |
| VAT | -0.0183073 | 0.00198982 | 3.60E-20 | rs862320 | T | C | 0.409765 | 84.64884043 |
| VAT | -0.0133722 | 0.00205068 | 7.00E-11 | rs12926311 | C | G | 0.353547 | 42.52163304 |
| VAT | -0.015738 | 0.00205102 | 1.70E-14 | rs28375268 | T | G | 0.645091 | 58.87884626 |
| VAT | -0.0132558 | 0.00242396 | 4.50E-08 | rs11075263 | T | C | 0.207814 | 29.90618312 |
| VAT | 0.0381119 | 0.00638813 | 2.40E-09 | rs117342986 | T | C | 0.026454 | 35.59374702 |
| VAT | 0.0110754 | 0.00196494 | 1.70E-08 | rs9673839 | G | A | 0.490953 | 31.77022226 |
| VAT | 0.0154078 | 0.00197492 | 6.10E-15 | rs12103006 | G | A | 0.569073 | 60.86704937 |
| VAT | -0.0113808 | 0.00199539 | 1.20E-08 | rs825680 | T | A | 0.413066 | 32.53044467 |
| VAT | 0.0112148 | 0.00197274 | 1.30E-08 | rs8076669 | C | T | 0.561582 | 32.31791726 |
| VAT | -0.0108198 | 0.00196298 | 3.50E-08 | rs8078135 | T | C | 0.48988 | 30.38132542 |
| VAT | 0.0347508 | 0.0038815 | 3.50E-19 | rs113866544 | C | T | 0.068327 | 80.15496576 |
| VAT | -0.0142713 | 0.00196663 | 4.00E-13 | rs35867081 | G | A | 0.512182 | 52.66010874 |
| VAT | -0.0272742 | 0.00271771 | 1.10E-23 | rs4790841 | T | C | 0.154491 | 100.7158487 |
| VAT | -0.0328285 | 0.00590379 | 2.70E-08 | rs117755721 | A | G | 0.02825 | 30.92005634 |
| VAT | -0.0151638 | 0.00199026 | 2.60E-14 | rs12937411 | T | C | 0.408044 | 58.04923037 |
| VAT | 0.0154995 | 0.00262729 | 3.60E-09 | rs17744603 | G | C | 0.16876 | 34.80321395 |
| VAT | 0.0269074 | 0.0024626 | 8.60E-28 | rs7218014 | C | T | 0.197323 | 119.3866372 |
| VAT | -0.0195604 | 0.00210446 | 1.50E-20 | rs11150745 | G | A | 0.31768 | 86.39211623 |
| VAT | 0.0135439 | 0.00208646 | 8.50E-11 | rs8074454 | C | G | 0.326764 | 42.13736273 |
| VAT | 0.0119493 | 0.00196225 | 1.10E-09 | rs3826408 | T | C | 0.456899 | 37.08311892 |
| VAT | 0.0179426 | 0.0028872 | 5.10E-10 | rs11870618 | A | G | 0.132541 | 38.6204218 |
| VAT | -0.0118759 | 0.0020105 | 3.50E-09 | rs35537311 | T | C | 0.388498 | 34.8919233 |
| VAT | 0.0143782 | 0.00206174 | 3.10E-12 | rs11656758 | G | A | 0.343119 | 48.63414072 |
| VAT | -0.0144978 | 0.00234319 | 6.10E-10 | rs11656076 | A | G | 0.224653 | 38.28152217 |
| VAT | 0.0120137 | 0.00208312 | 8.10E-09 | rs35937770 | A | G | 0.331139 | 33.26021011 |
| VAT | -0.0175566 | 0.002065 | 1.90E-17 | rs2052607 | A | G | 0.343856 | 72.28375697 |
| VAT | 0.0140652 | 0.00230384 | 1.00E-09 | rs11152135 | C | G | 0.760817 | 37.27238601 |
| VAT | 0.0178069 | 0.0027965 | 1.90E-10 | rs9955276 | T | C | 0.144424 | 40.54590442 |
| VAT | 0.0486668 | 0.00231465 | 3.80E-98 | rs6567160 | C | T | 0.23266 | 442.0739471 |
| VAT | -0.0148598 | 0.00233922 | 2.10E-10 | rs8093356 | T | C | 0.22697 | 40.35374001 |
| VAT | -0.0244406 | 0.00195957 | 1.10E-35 | rs1788808 | G | A | 0.494893 | 155.5615145 |
| VAT | 0.0143768 | 0.00215052 | 2.30E-11 | rs8096564 | T | G | 0.298122 | 44.69279244 |
| VAT | 0.0134456 | 0.00207285 | 8.80E-11 | rs11664848 | G | C | 0.659605 | 42.07504822 |
| VAT | -0.0378766 | 0.00354068 | 1.00E-26 | rs57636386 | C | T | 0.08384 | 114.4375679 |
| VAT | -0.0114957 | 0.00202787 | 1.40E-08 | rs1945160 | A | G | 0.375856 | 32.13591148 |
| VAT | 0.0135718 | 0.00218811 | 5.60E-10 | rs2276190 | A | G | 0.277449 | 38.47127682 |
| VAT | 0.0129327 | 0.00221726 | 5.50E-09 | rs2081880 | G | A | 0.382277 | 34.02085016 |
| VAT | -0.0192493 | 0.002003 | 7.20E-22 | rs8112818 | G | A | 0.400384 | 92.35660999 |
| VAT | -0.0116627 | 0.0019914 | 4.70E-09 | rs3764625 | G | T | 0.587569 | 34.29897986 |
| VAT | -0.0160421 | 0.00221757 | 4.70E-13 | rs12459965 | T | C | 0.267613 | 52.33205186 |
| VAT | -0.0132744 | 0.00235732 | 1.80E-08 | rs10421787 | T | C | 0.221235 | 31.70974274 |
| VAT | -0.0316258 | 0.00246865 | 1.40E-37 | rs10423928 | A | T | 0.194438 | 164.120941 |
| VAT | 0.0216824 | 0.00216059 | 1.10E-23 | rs2302209 | T | C | 0.288827 | 100.7093936 |
| VAT | 0.0178838 | 0.00230841 | 9.40E-15 | rs9304665 | A | T | 0.763666 | 60.01968541 |
| VAT | 0.0114484 | 0.00197346 | 6.60E-09 | rs12974458 | T | C | 0.543292 | 33.65370889 |
| VAT | -0.0190324 | 0.00251664 | 4.00E-14 | rs72976986 | A | G | 0.190201 | 57.19326936 |
| VAT | 0.0200333 | 0.00209576 | 1.20E-21 | rs12462975 | A | G | 0.329586 | 91.3738439 |
| VAT | -0.0170439 | 0.0019872 | 9.70E-18 | rs33836 | T | C | 0.464343 | 73.56221505 |
| VAT | -0.0263395 | 0.00271219 | 2.70E-22 | rs429358 | C | T | 0.154034 | 94.31371074 |
| VAT | 0.0161058 | 0.00196301 | 2.30E-16 | rs11698185 | C | T | 0.462588 | 67.31619806 |
| VAT | 0.0146479 | 0.00209635 | 2.80E-12 | rs6058209 | A | G | 0.32206 | 48.82285222 |
| VAT | 0.0154069 | 0.00209085 | 1.70E-13 | rs1056441 | C | T | 0.675302 | 54.29811753 |
| VAT | 0.020554 | 0.00293913 | 2.70E-12 | rs16996657 | C | T | 0.127763 | 48.9052083 |
| VAT | -0.0157344 | 0.0024087 | 6.50E-11 | rs56374036 | G | A | 0.212665 | 42.67120901 |
| VAT | -0.0250526 | 0.0024928 | 9.20E-24 | rs73142879 | T | C | 0.192216 | 101.0021775 |
| VAT | -0.0136445 | 0.00220742 | 6.40E-10 | rs6069037 | A | C | 0.731479 | 38.20720822 |
| VAT | 0.0127468 | 0.00210805 | 1.50E-09 | rs6029180 | G | A | 0.32599 | 36.56289105 |
| VAT | 0.0148532 | 0.00270269 | 3.90E-08 | rs74618095 | C | T | 0.157963 | 30.20282653 |
| VAT | -0.0133789 | 0.00216742 | 6.70E-10 | rs8132491 | A | G | 0.312976 | 38.10260444 |
| VAT | 0.0158642 | 0.00197101 | 8.40E-16 | rs394608 | C | T | 0.537787 | 64.78264822 |
| VAT | 0.0172096 | 0.00259365 | 3.20E-11 | rs11538 | G | A | 0.171827 | 44.02697152 |
| VAT | 0.0190883 | 0.00338986 | 1.80E-08 | rs9615937 | C | G | 0.092006 | 31.70815549 |
| VAT | -0.0193466 | 0.00198862 | 2.30E-22 | rs4820323 | G | C | 0.580959 | 94.6467486 |
| VAT | 0.0130604 | 0.001966 | 3.10E-11 | rs10854853 | T | G | 0.456743 | 44.13121958 |
| WHR | 0.028 | 0.0038 | 2.20E-13 | rs1106529 | A | G | 0.725 | 54.29362881 |
| WHR | 0.031 | 0.0044 | 1.70E-12 | rs1563355 | C | T | 0.6864 | 49.63842975 |
| WHR | -0.019 | 0.0033 | 5.90E-09 | rs714515 | A | G | 0.5417 | 33.1496786 |
| WHR | 0.025 | 0.0043 | 1.50E-08 | rs6743060 | A | C | 0.875 | 33.80205516 |
| WHR | -0.024 | 0.0042 | 7.20E-09 | rs6736025 | T | G | 0.4083 | 32.65306122 |
| WHR | -0.02 | 0.0033 | 7.80E-10 | rs1128249 | T | G | 0.4417 | 36.73094582 |
| WHR | -0.024 | 0.0032 | 2.60E-13 | rs13424740 | C | T | 0.4667 | 56.25 |
| WHR | -0.021 | 0.0034 | 1.20E-09 | rs17451107 | C | T | 0.375 | 38.14878893 |
| WHR | 0.019 | 0.0033 | 1.00E-08 | rs2972164 | C | T | 0.5 | 33.1496786 |
| WHR | -0.024 | 0.0037 | 1.20E-10 | rs2371767 | C | G | 0.2083 | 42.07450694 |
| WHR | 0.019 | 0.0033 | 1.50E-08 | rs13130484 | T | C | 0.4333 | 33.1496786 |
| WHR | -0.027 | 0.0037 | 3.40E-13 | rs459193 | G | A | 0.7833 | 53.25054785 |
| WHR | 0.025 | 0.0033 | 2.00E-14 | rs1294410 | C | T | 0.625 | 57.39210285 |
| WHR | 0.029 | 0.0036 | 4.80E-15 | rs998584 | A | C | 0.475 | 64.89197531 |
| WHR | 0.037 | 0.0032 | 1.40E-30 | rs9491696 | G | C | 0.525 | 133.6914063 |
| WHR | 0.027 | 0.0041 | 3.50E-11 | rs10245353 | A | C | 0.1833 | 43.36704343 |
| WHR | 0.037 | 0.006 | 8.30E-10 | rs12549058 | G | T | 0.0583 | 38.02777778 |
| WHR | -0.02 | 0.0036 | 4.00E-08 | rs2398893 | G | A | 0.3167 | 30.86419753 |
| WHR | 0.019 | 0.0033 | 1.60E-08 | rs4929927 | G | A | 0.725 | 33.1496786 |
| WHR | 0.022 | 0.004 | 3.60E-08 | rs879048 | A | C | 0.8 | 30.25 |
| WHR | -0.034 | 0.0046 | 3.70E-13 | rs10783615 | A | G | 0.8667 | 54.63137996 |
| WHR | -0.024 | 0.0036 | 3.10E-11 | rs10842708 | A | G | 0.8167 | 44.44444444 |
| WHR | -0.02 | 0.0034 | 3.30E-09 | rs7973683 | A | C | 0.3833 | 34.60207612 |
| WHR | 0.022 | 0.0036 | 2.70E-09 | rs1440372 | C | T | 0.7417 | 37.34567901 |
| WHR | 0.043 | 0.0033 | 4.30E-38 | rs1421085 | C | T | 0.45 | 169.7887971 |
| WHR | 0.02 | 0.0037 | 3.00E-08 | rs4640244 | G | A | 0.375 | 29.2184076 |
| WHR | 0.026 | 0.0037 | 3.10E-12 | rs11663816 | C | T | 0.3167 | 49.37910884 |
| WHR | 0.022 | 0.0033 | 2.50E-11 | rs3786897 | G | A | 0.4083 | 44.44444444 |
| WHR | -0.025 | 0.0044 | 1.10E-08 | rs2287019 | T | C | 0.15 | 32.28305785 |
| WHR | -0.021 | 0.0036 | 8.80E-09 | rs16996700 | C | T | 0.3 | 34.02777778 |
| WHR | -0.019 | 0.0033 | 3.30E-09 | rs4823006 | G | A | 0.4667 | 33.1496786 |
